# Relation Between Dietary Carotenoid Intake, Serum Concentration, and Mortality Risk of CKD Patients Among US Adults: National Health and Nutrition Examination Survey 2001–2014

**DOI:** 10.3389/fmed.2022.871767

**Published:** 2022-07-08

**Authors:** Yuncan Hu, Xiaoyu Cai, Nanhui Zhang, Yu Li, Ya Mao, Shuwang Ge, Ying Yao, Hui Gao

**Affiliations:** ^1^Department of Nephrology, Tongji Medical College, Tongji Hospital, Huazhong University of Science and Technology, Wuhan, China; ^2^Department of Nephrology, Xiangyang No.1 Peoples Hospital Affiliated Hospital of Hubei University of Medicine, Xiangyang, China; ^3^Department of Clinical Nutrition, Tongji Medical College, Tongji Hospital, Huazhong University of Science and Technology, Wuhan, China

**Keywords:** chronic kidney disease, carotenoid, mortality, dietary intake, NHANES

## Abstract

**Background:**

Current evidence on the relationship between carotenoids and chronic kidney disease (CKD) patients are limited and controversial.

**Methods:**

Data were obtained from the Nutrition and Health Examination Survey (NHANES) database and the NHANES Linked Mortality File, both from a nationally representative sample. Dietary intake was assessed through 24-h dietary recall, and information was available both on dietary and serum α-carotene, β-carotene, β-cryptoxanthin, lycopene, and lutein/zeaxanthin (combined) through the NHANES cycles used. We used multivariable Cox proportional hazards regression models to estimate the risk for all-cause mortality associated with carotene intakes and serum levels, adjusting for potential confounding factors.

**Results:**

Of the 6,095 CKD participants, 1,924 subjects died (mean follow-up time, 8.1 years). After eliminating all the confounding factors, we found that high levels of total carotene (HR = 0.85, 95% CI, 0.75-0.97, P = 0.011) intakes at baseline were significantly associated with a lower risk of death. And the serum concentrations of carotenoid were also showing that a-carotene (HR = 0.77, 95%CI, 0.65–0.92, *P* = 0.002), beta-cryptoxanthin (HR = 0.83, 95%CI, 0.70–0.98, *P* = 0.019), lycopene (HR = 0.77, 95% CI, 0.65–0.91, P = 0.002), and lutein + zeaxanthin (HR = 0.82, 95% CI, 0.70–0.96, P = 0.002) was significantly associated with decreased all-cause mortality of CKD patients. The associations remained similar in the sensitivity analyses.

**Conclusion:**

Findings suggest that high-level carotene dietary intake and the serum concentration were associated with a lower risk of mortality in the CKD population.

## Introduction

Chronic kidney disease (CKD) is a major problem that threatens global public health with an increasing incidence and prevalence, while 697.5 million cases of CKD globally when the prevalence of CKD was estimated as 9.1% in 2017 (1–3). And the CKD patients, which can progress to end-stage renal disease (ESRD) and become a major contributor to cardiovascular death, has been reported that the mortality rate is much higher than people without CKD (1–4). However, there are still not many effective strategies that can slow the progression of CKD. To improve the survival in CKD, a great many studies conducted and nutritional supplementation has been considered as a direction of treatment (5).

It is known to all that CKD is a chronic inflammatory disease that involves oxidative stress, which is thought to be the key factor in the progression of CKD (6). Though the precise mechanisms have not been elucidated yet, oxidative stress characterized by an imbalance between the accumulation of reactive oxygen species (ROS) and the natural ability of anti-oxidant in the cell occurs frequently in CKD (7–9). Furthermore, oxidative stress also plays a vital role in the conditions of cancer, cardiovascular disease, diabetes, hypertension, and infection, which are known to be strongly associated with mortality in patients with CKD (7, 8).

As powerful antioxidants, carotenoids can remove ROS and enhance the cell's ability to prevent oxidative stress to delay the progression of the disease, which is considered as an emerging therapeutic direction in CKD patients (10). It can be classified into two major types: carotenes and xanthophylls. Carotenes, which include β-carotene, α-carotene, and lycopene as well as other less-studied species, are unoxygenated terpenes, whereas xanthophylls, which include lutein, zeaxanthin, and β-cryptoxanthin, are oxygenated (11). All these carotenoids have been verified that have their unique antioxidant properties (12) and can scavenge radicals in three steps such as electron transfer, hydrogen abstraction, and addition, to act as the main scavenger of the ROS (12). Previous studies have shown that carotenoids are associated with decreased all-cause mortality and lung cancer mortality in US adults (13, 14). Moreover, researchers also found that serum carotenoids were associated with estimated glomerular filtration rate (eGFR), both in an aged cohort and preserved kidney function patients (15, 16). And there are animal studies to support that the β-carotene possesses a nephroprotective effect through bromobenzene-administered rats (17, 18). However, whether the carotenoid intake was associated with the mortality of CKD patients hasn't been found yet.

Therefore, this study aims to characterize whether carotenoid intake was associated with the mortality risk of CKD patients, while data was obtained from the Nutrition and Health Examination Survey (NHANES) database and the NHANES Linked Mortality File.

## Materials and Methods

### Study Design and Population

Data were screened out of the NHANES database from 2001 to 2014, which is a periodic survey conducted by the National Center for Health Statistics (NCHS) of the Centers for Disease Control and Prevention (CDC) (19). And the NHANES is a complex, multistage probability sampling design using a nationally representative sample of the non-institutionalized civilian population of the USA. All participants were selected randomly through a complex statistical process each year. They complete personal structured interviews at home and then undergo a physical examination at a mobile examination central, including height, weight, laboratory measurements, and a computer-assisted 24-h dietary recall (20). Both ethics approval and data collection for NHANES were obtained from the NCHS Research Ethics Review Board and written informed consent was provided by every participant. More details are available on the NHANES website (19).

**Figure 1:**
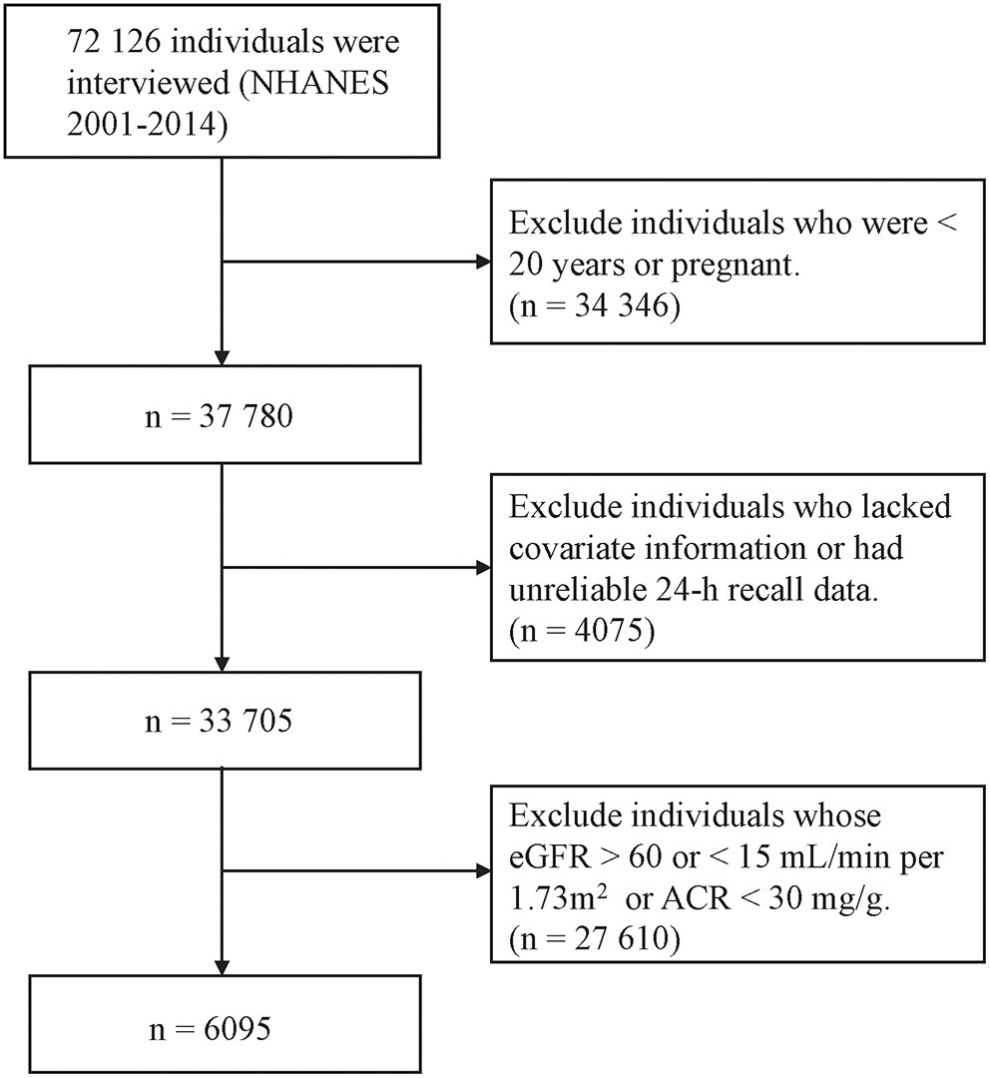
Flow diagram of the selection of eligible participants, National Health and Nutrition Examination Survey 2001–2014.

Eligibility criteria included CKD participants with age ≥ 20 years old and nonpregnant. CKD was defined as an estimated glomerular filtration rate (eGFR) <60 mL/min/1.73 m^2^ (using the Chronic Kidney Disease Epidemiology Collaboration equation) and/or urinary albumin: creatinine ratio >30 mg/g (1). To assess the quality and completeness of a survey participant's response to the dietary recall section, we used the dietary recall status evaluated by the interviewer and only included the ones that were classified as ‘reliable and met the minimum criteria. Finally, 6,313 participants with complete data were analyzed (Figure 1).

### Assessments of Dietary Intakes and Serum Levels of Carotenoids

In all cycles of the NHANES 2001–2014, dietary intakes were estimated using data from twice 24-h dietary recalls (2003–2014), or only one (2001–2002). As for the primary dietary interview, one was conducted in person at the mobile examination center, and the other was conducted by telephone after 3–10 days. All interviews were carried out by trained investigators according to the US Department of Agriculture Automated Multiple-Pass Method for the 24-h recall (21, 22). According to previous analyses, we used the nutritional information from foods and beverages collected in the single 24-h dietary recall to calculate the carotenes, energy, and nutrient intakes of participants in the 2001–2002 NHANES. And for participants in the 2003–14 NHANES, we used the mean of the nutritional information from both recalls.

As for the measurements of serum carotenoids, the information of part participants was available on α-carotene, β-carotene, β-cryptoxanthin, lycopene, and lutein/zeaxanthin (combined). In these cycles, participants aged more than 6 years provided serum samples for measurement of six carotenoids (α-carotene, trans-β-carotene, cis β-carotene, β-cryptoxanthin, combined lutein/zeaxanthin, trans-lycopene, and total lycopene) using high-performance liquid chromatography (HPLC) (23). And we evaluated serum levels of carotenoids to CKD outcomes as a sub-analysis.

### Assessments of Other Variables

Based on the existing literature, the following variables were selected as confounding factors measured in the baseline survey. The demographic characteristics included age (years), sex (men or women), race/ethnicity, education level, and marital status (24). Lifestyle-related behaviors included leisure-time physical activity, smoking status, and alcohol consumption status (25). The details of the data collection and definition have been described in detail elsewhere.

Blood specimen collections and measurements of blood pressure, body weight, and height were conducted during mobile physical examinations. Body mass index (BMI) was calculated as body weight (kg) divided by the square of height (m^2^). Three consecutive blood pressure readings were obtained after resting quietly in a sitting position for 5 min, and the means of readings were calculated (19). Moreover, the fasting plasma glucose, glycosylated hemoglobin, blood total cholesterol, HDL level, urine albumin, urine creatinine (Cr), serum creatinine, serum phosphorus, and hemoglobin were measured in the same laboratory, where detail was available on the NHANES website (19). More particularly, the serum Cr was measured using a recalibration equations for 1999–2000 and 2005–2006 NHANES surveys: standard serum Cr (mg/dL) = 0.147 + 1.013 × uncalibrated serum Cr (mg/dL) and standard serum Cr (mg/dL) = −0.016 + 0.978 × uncalibrated serum Cr (mg/dL) (26). And Serum Cr-based eGFR was estimated using the CKD Epidemiology Collaboration equation.

Diabetes was defined that reported: (1) self-reported diabetes; (2) the use of anti-hyperglycemic agents; (3)measured fasting plasma glucose ≥ 126 mg/dL; (4) 2-h plasma glucose ≥ 200 mg/dL during a 75-g oral glucose tolerance test; (5) glycol-hemoglobin ≥ 6.5% (27). And hypertension was defined as people who: (1) self-reported hypertension; (2) reported use of antihypertensive agents; (3) measured systolic blood pressure ≥ 140 mmHg; (4)diastolic blood pressure ≥ 90 mmHg (27). High cholesterol levels were defined as the total-to-HDL cholesterol ratio was more than 5.9 (27). And participants were considered to have a history of cardiovascular disease if they reported ever being told they had any of the following conditions by a health care provider: congestive heart failure, coronary heart disease, angina pectoris, heart attack, or stroke (28).

### Outcome Assessment

The study's endpoint was the follow-up to December 31, 2015, or death. If the patient had died before December 31, 2015, the time of death would be the end time of follow-up. The National Center for Health Statistics (NCHS) has linked data collected from NHANES with death certificate records from the National Death Index (NDI) with a probabilistic matching algorithm and social security number, name, father's surname, birthday, state of birth, state of residence, sex, race, and marital status were used to match NHANES records with National Death Index records. The International Statistical Classification of Diseases, 10th Revision was used to identify the causes of deaths, like previous studies (29–31).

### Statistical Analysis

Baseline characteristics were described across the groups, according to intakes of carotenes, and between-group differences were tested by analysis of variance for continuous variables and Rao-Scott χ^2^ test for categorical variables. Multivariable Cox proportional hazards regression models were used to estimating the risk for all-cause mortality associated with carotene intakes, adjusting for potential confounding factors including demographic factors, lifestyle behaviors, history of chronic health conditions, and dietary factors. We used the Schoenfeld residual plots to examine the proportional hazards assumption, and no violations were noted. We adjusted for age (years, continuous), sex, family income-poverty ratio (≤ 1, > 1 to <4, and ≥ 4), self-reported race (non-Hispanic white, non-Hispanic black, Mexican American, and others), an education level (less than high school graduates, high school graduates or equivalent, and college or above), marital status (married and not married), alcohol consumption (none, ≤ 2 drinks/d for men or ≤ 1 drink/d for women, 2–5 drinks/d for men or 1–4 drink/d for women, > 5 drinks/d for men or >4 drink/d for women), cigarette smoking (never, former, current), and vigorous/moderate recreational activities for at least 10 min continuously per week (%) in model 1, log-transformed total energy intake (kilocalorie, continuous), HEI2015, baseline eGFR, log-transformed urinary ACR, BMI (kg/m^2^, continuous), high cholesterol levels, serum phosphorus, hemoglobin, hypertension, and diabetes, and history of CVD and cancer were additionally adjusted in model 2. Restricted cubic splines (RCSs) were applied to examine the possible nonlinear relationship between intakes of carotenes (as continuous variables) and mortality, using the median intakes of each group as knots (32).

To assess whether the association of SSB with all-cause mortality was different because of the demographic characteristics and life behavioral habits of CKD patients, stratified analyses was conducted using a Wald test according to age (<60 vs. ≥ 60 years), sex, self-reported race (non-Hispanic white vs. minority ethnic groups), BMI (<25 vs. ≥ 25 kg/ m^2^), history of hypertension, diabetes, CVD, or cancer, CKD stage, serum phosphorus and hemoglobin level. A cross-product term between dietary intakes and each grouping variable was correspondingly added to the model, the likelihood ratio test was used to evaluate if there was a statistically significant interaction. And several sensitivity analyses were conducted. First, we repeated the main analysis by replacing the HEI-2015 with intakes of major food groups (i.e., vegetable, fruit, whole grain, red meat, and processed meat) or macronutrients. Second, we repeated the main analysis by adjusting for waist circumference instead of BMI. Third, concerning previous studies, we excluded adults who died within 1 year after the survey to minimize potential reverse causation. Simple correlation analyses were performed using Pearson correlation to assess associations between carotene intakes and its correspondence serum level. Hazards ratios (HRs) and 95% confidence intervals (CIs) were reported (33). A 2-sided *p* < 0.05 was considered statistically significant. Multiple imputations by chained equations were used for dealing with missing data regarding covariates (34). All data management and analyses were performed in RStudio statistical software (version 1.1.423).

## Results

Table 1 compares the characteristics of the study participants which are divided into four quartiles. Of 6,095 CKD participants, 1,924 subjects died (mean follow-up time, 8.1 years). Compared with the lower quartiles, the upper quartiles group of carotenoid concentrations was more likely to be patients who were non-Hispanic whites, married, higher educated, higher family income-poverty ratio level, moderate drinker, never smoking, more active, higher dietary acid load, and cancer. And it also has the characteristics of more plurality prevalent cases of non-drinkers, current-smokers, diabetes, hypertension, and history of CVD in the group of lower serum carotenoid. Furthermore, the people with higher serum carotenoid concentration seem to have lower BMI, total energy intake, HEI-2015, dietary acid load, and eGFR.

**Table 1:** Baseline characteristics of participants by quartiles of total carotenoids, NHANES, 2001–2014.

**Characteristics**		**Total carotenoids quartile (mcg/kg/d)**				***P***
		**Quartile 1**	**Quartile 2**	**Quartile 3**	**Quartile 4**	
	**Missing (%)**	**≦29.91**	**29.92–69.91**	**69.92–141.89**	**≧141.90**	
N (proportion in the total population)		1,523 (25)	1,525 (25)	1,524 (25)	1,523 (25)	-
Age, years [mean (SD)]	0 (0)	64.26 (16.11)	64.67 (16.15)	64.22 (16.85)	64.57 (17.29)	0.834
Male (%)	0 (0)	754 (49.5)	721 (47.3)	706 (46.3)	715 (46.9)	0.318
Self-reported race/ethnicity (%)	0 (0)					**<0.001**
Mexican American	-	181 (11.9)	236 (15.5)	226 (14.8)	183 (12.0)	-
Others	-	159 (10.4)	158 (10.4)	190 (12.5)	191 (12.5)	-
Non-Hispanic white	-	726 (47.7)	819 (53.7)	826 (54.2)	861 (56.5)	-
Non-Hispanic black	-	457 (30.0)	312 (20.5)	282 (18.5)	288 (18.9)	-
Married (%)	0 (0)	682 (44.8)	790 (51.8)	759 (49.8)	791 (51.9)	**<0.001**
Education level (%)	0 (0)					**<0.001**
Less than high school	-	662 (43.5)	573 (37.6)	472 (31.0)	436 (28.6)	-
High school graduates or equivalent	-	369 (24.2)	368 (24.1)	390 (25.6)	340 (22.3)	-
Some College or above	-	492 (32.3)	584 (38.3)	662 (43.4)	747 (49.0)	-
Family income-poverty ratio level (%)	472 (7.7)					**<0.001**
≥ 4	-	198 (13.0)	276 (18.1)	301 (19.8)	343 (22.5)	-
> 1 to <4	-	969 (63.6)	946 (62.0)	944 (61.9)	943 (61.9)	-
≤ 1	-	356 (23.4)	303 (19.9)	279 (18.3)	237 (15.6)	-
Alcohol drinking (%)	370 (6.1)					**<0.001**
Non-drinkers	-	592 (38.9)	597 (39.1)	538 (35.3)	508 (33.4)	-
Moderate drinkers	-	543 (35.7)	553 (36.3)	600 (39.4)	660 (43.3)	-
Binge drinkers	-	251 (16.5)	252 (16.5)	242 (15.9)	231 (15.2)	-
Heavy drinkers	-	137 (9.0)	123 (8.1)	144 (9.4)	124 (8.1)	-
Cigarette smoking (%)	5 (0.1)					**<0.001**
Never smoking	-	703 (46.2)	736 (48.3)	737 (48.4)	804 (52.8)	-
Former smoking	-	510 (33.5)	528 (34.6)	538 (35.3)	508 (33.4)	-
Current smoking	-	310 (20.4)	261 (17.1)	249 (16.3)	211 (13.9)	-
Vigorous/moderate recreational activities for at least 10 min continuously per week (%)	259 (4.2)	504 (33.1)	580 (38.0)	636 (41.7)	696 (45.7)	**<0.001**
Body mass index, kg/m^2^ (%)	209 (3.4)					**<0.001**
<18.5	-	19 (1.2)	19 (1.2)	27 (1.8)	48 (3.2)	-
18.5–24.9	-	300 (19.7)	298 (19.5)	329 (21.6)	518 (34.0)	-
25.0–29.9	-	470 (30.9)	498 (32.7)	559 (36.7)	511 (33.6)	-
≥30.0	-	734 (48.2)	710 (46.6)	609 (40.0)	446 (29.3)	-
Total-to-HDL cholesterol ratio ≥5.9 (%)	238 (3.9)	174 (11.4)	169 (11.1)	143 (9.4)	119 (7.8)	**0.003**
Prevalent hypertension (%)	245 (4.0)	1,148 (75.4)	1,125 (73.8)	1,098 (72.0)	1,040 (68.3)	**<0.001**
Prevalent diabetes (%)	231 (3.8)	462 (30.3)	466 (30.6)	459 (30.1)	358 (23.5)	**<0.001**
History of CVD (%)	57 (0.9)	481 (31.6)	446 (29.2)	420 (27.6)	408 (26.8)	**0.018**
History of cancer (%)	11 (0.2)	241 (15.8)	242 (15.9)	268 (17.6)	281 (18.5)	0.138
CKD stage (%)	255 (4.2)					**0.001**
1	-	386 (25.3)	378 (24.8)	397 (26.0)	426 (28.0)	-
2	-	357 (23.4)	344 (22.6)	356 (23.4)	353 (23.2)	-
3	-	671 (44.1)	699 (45.8)	702 (46.1)	687 (45.1)	-
4–5	-	109 (7.2)	104 (6.8)	69 (4.5)	57 (3.7)	-
Dietary Measures						
Energy intake, kcal/d, (median [IQR])	0 (0)	1,443.00 [1,067.25, 1,935.00]	1,590.50 [1,260.50, 2,080.00]	1,735.25 [1,351.12, 2,222.62]	1,849.00 [1,442.50, 2,364.50]	**<0.001**		**Quartile 1**	**Quartile 2**	**Quartile 3**	**Quartile 4**	
	**Missing (%)**	**≦29.91**	**29.92–69.91**	**69.92–141.89**	**≧141.90**	
HEI-2015 [mean (SD)]	44 (0.7)	48.56 (11.59)	52.74 (12.76)	54.36 (12.60)	56.44 (13.39)	**<0.001**
Dietary acid load, mEq/d [mean (SD)]	0 (0)	12.45 (16.56)	11.56 (17.53)	10.31 (20.31)	5.07 (19.21)	**<0.001**
Alpha-carotene (mcg/kg per day) (median [IQR])	-	0.25 [0.06, 0.61]	1.08 [0.37, 4.18]	2.39 [0.59, 8.86]	3.98 [0.76, 13.82]	**<0.001**
Beta-carotene (mcg/kg per day) (median [IQR])	-	2.78 [1.42, 5.19]	10.96 [5.72, 19.45]	24.18 [10.33, 41.81]	46.53 [19.86, 90.17]	**<0.001**
Beta-cryptoxanthin (mcg/kg per day) (median [IQR])	-	0.20 [0.06, 0.82]	0.54 [0.17, 1.52]	0.86 [0.24, 2.08]	1.04 [0.34, 2.70]	**<0.001**
Lycopene (mcg/kg per day) (median [IQR])	-	0.34 [0.00, 7.39]	17.85 [5.35, 30.57]	45.89 [14.19, 74.03]	127.74 [44.88, 199.96]	**<0.001**
Lutein + zeaxanthin (mcg/kg per day) (median [IQR])	-	4.70 [2.42, 7.45]	8.80 [5.33, 14.11]	12.78 [7.14, 23.33]	19.61 [9.94, 48.44]	**<0.001**
eGFR, ml/min per 1.73 m^2^ [mean (SD)]	179 (2.9)	69.64(0.75)	68.80(0.74)	70.73(0.71)	71.83(0.71)	**0.013**
ACR, mg/g (median [IQR])	255 (4.2)	46.43 [16.43–115.38]	40.19 [11.41–109.85]	40.51 [14.11–87.95]	40.12 [11.62–94.51]	0.384
Serum phosphorus, mg/dL [median (SD)]	255 (4.2)	3.76 (0.68)	3.77 (0.60)	3.78 (0.58)	3.81 (0.59)	0.199
Hemoglobin, g/dL [mean (SD)]	178 (2.9)	13.67 (1.74)	13.73 (1.72)	13.78 (1.60)	13.79 (1.68)	0.196
Serum Measures (2001–2006 only)						
Serum α-carotene (μg/dL) (median [IQR])	-	2.10 [1.10, 3.75]	2.80 [1.50, 5.21]	3.05 [1.66, 5.40]	3.50 [1.90, 6.60]	**<0.001**
Serum β-carotene (μg/dL) (median [IQR])	-	11.89 [6.80, 21.21]	15.40 [8.39, 27.70]	15.57 [9.18, 26.62]	19.05 [10.30, 33.88]	**<0.001**
Serum β-cryptoxanthin (μg/dL) (median [IQR])	-	6.60 [4.03, 10.40]	8.30 [4.82, 13.26]	8.96 [5.59, 13.40]	9.53 [5.80, 15.60]	**<0.001**
Serum lycopene (μg/dL) (median [IQR])	-	19.87 [11.71, 31.35]	25.00 [14.18, 37.71]	28.74 [18.30, 41.25]	28.28 [17.92, 45.20]	**<0.001**
Serum lutein/zeaxanthin (μg/dL) (median [IQR])	-	12.80 [9.20, 17.70]	14.30 [10.46, 20.20]	14.94 [10.26, 21.30]	17.00 [12.20, 23.80]	**<0.001**

*Categorical variables were given as number (percentage), and continuous variables as median with interquartile range due to their skewed distributions.*

*NHANES, National Health and Nutrition Examination Survey; SD, standard deviation; IQR, interquartile range; HDL, high density lipoprotein; CVD, cardiovascular disease; CKD, chronic kidney disease; HEI-2015, healthy eating index score-2015; eGFR, estimated glomerular filtration rate; ACR, albumin-Cr ratio.*

*Statistically significant results are shown in bold*.

Table 2 shows the baseline carotenoid intake levels of the total and lycopene were associated with the low mortality in CKD patients. When adjustments were made for model 1 (age, sex, family income-poverty ratio level, race, education level, marital status, alcohol consumption, smoking, and leisure-time physical activity), the risk of death was remain reduced in participants with higher total carotene (HR = 0.82, 95% CI, 0.72-0.93, *P* = 0.003), β-carotene intake (HR = 0.82, 95%CI, 0.73–0.94, *P* = 0.005) and lycopene intake (HR = 0.84, 95%CI, 0.74–0.95, *P*=0.01)levels at baseline than in those with the lowest levels. And when additionally adjusted for model 2 (model 1 plus log-transformed total energy intake, HEI-2015, baseline eGFR, log-transformed urinary ACR, body mass index, total-to-HDL cholesterol ratio, serum phosphorus, hemoglobin, hypertension, diabetes, and history of cardiovascular disease and cancer), the significant differences of the total carotene(HR = 0.85, 95% CI, 0.75-0.97, *P* = 0.011), β-carotene (HR = 0.86, 95%CI, 0.76–0.99, *P* = 0.007) as well as lycopene intake (HR = 0.85, 95% CI, 0.75–0.97, *P* = 0.028) are still existing in CKD persons. The dose-response analysis of the associations of daily intakes of carotene with mortality shows the same results, and a non-linear relationship was not indicated in all kinds of carotenoids (Supplementary Figure 1). However, results were substantially changed when we further adjusted for substituting HEI-2015 by food groups, substituting HEI-2015 by macronutrients, waist circumference instead of BMI, or excluding those who died within 1 year (Table 3). The intake of total carotene always has a significant association with the death of CKD patients while the β-carotene and lycopene were not.

**Table 2:** The associations of daily intakes of carotene with mortality.

	**Cutoff**	**Cases/participants**	**Incidence Rate per 1,000 Person-Years (95% CI)**	**Crude**	**Model 1**	**Model 2**
				**HR (95% CI)**	**HR (95% CI)**	**HR (95% CI)**
**Alpha-carotene intake (mcg/kg per day)**						
Quartile 1	≦0.29	511/1,527	51.6 (47.4–56.2)	ref	ref	ref
Quartile 2	0.30–1.05	466/1,531	46.0 (42.0–50.3)	0.89 (0.79–1.01)	**0.88 (0.77–0.99)**	0.91 (0.80–1.04)
Quartile 3	1.06–5.53	462/1,519	48.3 (44.1–52.8)	0.94 (0.83–1.07)	**0.84 (0.74–0.96)**	0.87 (0.76–0.99)
Quartile 4	At least 5.54	485/1,518	50.2 (46.0–54.8)	0.98 (0.87–1.11)	0.91 (0.80–1.03)	0.95 (0.83–1.09)
P trend				0.967	0.155	0.481
**Beta-carotene intake (mcg/kg per day)**						
Quartile 1	≦4.90	498/1,525	50.6 (46.4–55.2)	ref	ref	ref
Quartile 2	4.91–13.16	453/1,523	46.6 (42.5–51.0)	0.92 (0.81–1.05)	**0.86 (0.76–0.98)**	0.90 (0.79–1.02)
Quartile 3	13.17–34.21	479/1,524	48.8 (44.7–53.3)	0.97 (0.85–1.10)	**0.86 (0.75–0.97)**	0.88 (0.77–1.00)
Quartile 4	At least 34.22	494/1,523	50.0 (45.8–54.5)	0.99 (0.87–1.12)	**0.82 (0.73–0.94)**	**0.86 (0.76–0.99)**
P trend				0.963	**0.005**	**0.007**
**Beta-cryptoxanthin intake (mcg/kg per day)**						
Quartile 1	≦0.15	450/1,532	44.1 (40.2–48.3)	ref	ref	ref
Quartile 2	0.16–0.60	399/1,523	42.7 (38.7–47.0)	0.99 (0.86–1.13)	0.98 (0.85–1.12)	1.00 (0.87–1.15)
Quartile 3	0.61–1.77	473/1,523	**52.3 (47.8–57.1)**	**1.21 (1.07–1.38)**	1.11 (0.97–1.26)	**1.15 (1.01–1.32)**
Quartile 4	At least 1.78	602/1,517	**56.4 (52.1–61.0)**	**1.27 (1.12–1.43)**	1.07 (0.95–1.21)	**1.15 (1.00–1.31)**
P trend				**<0.001**	0.129	**0.013**
**Lycopene intake (mcg/kg per day)**						
Quartile 1	≦2.43	612/1,525	62.2 (57.5–67.2)	ref	ref	ref
Quartile 2	2.44–20.19	457/1,523	**47.6 (43.5–52.1)**	**0.77 (0.68–0.87)**	0.91 (0.80–1.03)	0.92 (0.81–1.04)
Quartile 3	20.20–67.59	435/1,524	**45.1 (41.1–49.5)**	**0.73 (0.65–0.83)**	0.92 (0.81–1.04)	0.96 (0.84–1.08)
Quartile 4	At least 67.60	420/1,523	**41.3 (37.6–45.4)**	**0.66 (0.59–0.75)**	**0.84 (0.74–0.95)**	**0.85 (0.75–0.97)**
P trend				**<0.001**	**0.01**	**0.028**
**Lutein** **+** **zeaxanthin intake (mcg/kg per day)**						
Quartile 1	≦5.06	483/1,525	48.0 (43.9–52.4)	ref	ref	ref
Quartile 2	5.07–9.51	458/1,523	47.0 (42.9–51.4)	0.99 (0.87–1.12)	0.91 (0.80–1.04)	0.96 (0.84–1.10)
Quartile 3	9.52–18.47	497/1,523	52.2 (47.9–56.9)	1.10 (0.97–1.24)	0.98 (0.87–1.12)	1.04 (0.91–1.19)
Quartile 4	At least 18.48	486/1,524	48.9 (44.8–53.4)	1.02 (0.90–1.16)	0.90 (0.79–1.02)	0.97 (0.84–1.12)
P trend				0.408	0.245	0.942
**Total carotene intake (mcg/kg per day)**						
Quartile 1	≦29.91	531/1,523	54.8 (50.4–59.6)	ref	ref	ref
Quartile 2	29.92–69.91	475/1,525	**50.1 (45.8–54.7)**	0.92 (0.81–1.04)	0.94 (0.83–1.06)	0.98 (0.86–1.11)
Quartile 3	69.92–141.89	473/1,524	**47.4 (43.4–51.8)**	**0.86 (0.76–0.98)**	0.91 (0.80–1.03)	0.92 (0.81–1.04)
Quartile 4	At least 141.90	445/1,523	**44.0 (40.1–48.2)**	**0.80 (0.71–0.91)**	**0.82 (0.72–0.93)**	**0.85 (0.75–0.97)**
P trend				**<0.001**	**0.003**	**0.011**

*Model 1 adjusted for age, sex, family income-poverty ratio level, race, education level, marital status, alcohol consumption, smoking, and leisure-time physical activity.*

*Model 2 additionally adjusted for log-transformed total energy intake, HEI-2015, baseline eGFR, log-transformed urinary ACR, body mass index, total-to-HDL cholesterol ratio, hypertension, serum phosphorus, hemoglobin, diabetes, and history of cardiovascular disease and cancer.*

*HR, hazard ratio, serum phosphorus, hemoglobin; CI, confidence interval; HDL, high density lipoprotein; HEI-2015, healthy eating index score-2015; eGFR, estimated glomerular filtration rate; ACR, albumin-Cr ratio.*

*Statistically significant results are shown in bold*.

**Table 3:** Results of sensitivity analyses of the associations between carotene intakes and mortality.

	**Q1**	**Q2**	**Q3**	**Q4**	**P trend**
**Substituting HEI-2015 by food groups**				
Alpha-carotene (mcg/kg per day)	ref	0.89 (0.79–1.02)	0.85 (0.75–0.97)	0.92 (0.81–1.05)	0.151
Beta-carotene (mcg/kg per day)	ref	0.88 (0.78–1.00)	0.86 (0.76–0.98)	0.84 (0.74–0.95)	**0.008**
Beta-cryptoxanthin (mcg/kg per day)	ref	0.98 (0.85–1.12)	1.11 (0.98–1.27)	1.10 (0.97–1.25)	0.075
Lycopene (mcg/kg per day)	ref	0.92 (0.82–1.05)	0.95 (0.84–1.08)	0.86 (0.75–0.97)	**0.031**
Lutein + zeaxanthin (mcg/kg per day)	ref	0.95 (0.84–1.09)	1.02 (0.89–1.16)	0.94 (0.82–1.08)	0.280
Total carotene intake (mcg/kg per day)	ref	0.96 (0.85–1.09)	0.90 (0.80–1.02)	0.83 (0.73–0.95)	**0.003**
**Substituting HEI-2015 by macronutrients**				
Alpha-carotene (mcg/kg per day)	ref	0.89 (0.79–1.02)	0.85 (0.75–0.97)	0.92 (0.81–1.05)	0.151
Beta-carotene (mcg/kg per day)	ref	0.88 (0.78–1.00)	0.86 (0.76–0.98)	0.84 (0.74–0.95)	**0.008**
Beta-cryptoxanthin (mcg/kg per day)	ref	0.98 (0.85–1.12)	1.11 (0.98–1.27)	1.10 (0.97–1.25)	0.075
Lycopene (mcg/kg per day)	ref	0.92 (0.82–1.05)	0.95 (0.84–1.08)	0.86 (0.75–0.97)	**0.031**
Lutein + zeaxanthin (mcg/kg per day)	ref	0.96 (0.84–1.09)	1.02 (0.89–1.16)	0.94 (0.82–1.07)	0.280
Total carotene intake (mcg/kg per day)	ref	0.96 (0.85–1.09)	0.90 (0.80–1.02)	0.83 (0.73–0.95)	**0.003**
**Adjusting for waist circumference instead of BMI**			
Alpha-carotene (mcg/kg per day)	ref	0.92 (0.81–1.05)	0.89 (0.78–1.01)	0.97 (0.85–1.11)	0.529
Beta-carotene (mcg/kg per day)	ref	0.90 (0.79–1.02)	0.89 (0.78–1.02)	0.89 (0.77–1.01)	0.081
Beta-cryptoxanthin (mcg/kg per day)	ref	0.99 (0.86–1.13)	1.13 (0.99–1.29)	1.12 (0.99–1.28)	**0.013**
Lycopene (mcg/kg per day)	ref	0.92 (0.81–1.04)	0.95 (0.84–1.08)	0.86 (0.75–0.97)	**0.032**
Lutein + zeaxanthin (mcg/kg per day)	ref	0.97 (0.85–1.10)	1.06 (0.92–1.21)	1.01 (0.88–1.17)	0.895
Total carotene intake (mcg/kg per day)	ref	0.99 (0.87–1.12)	0.93 (0.82–1.05)	0.87 (0.76–0.99)	**0.025**
**Excluding those died within one year**				
Alpha-carotene (mcg/kg per day)	ref	0.88 (0.77–1.00)	0.84 (0.73–0.96)	0.91 (0.79–1.04)	0.302
Beta-carotene (mcg/kg per day)	ref	0.87 (0.76–1.00)	0.85 (0.74–0.97)	0.84 (0.73–0.95)	**0.009**
Beta-cryptoxanthin (mcg/kg per day)	ref	0.96 (0.83–1.11)	1.12 (0.98–1.29)	1.11 (0.97–1.27)	**0.040**
Lycopene (mcg/kg per day)	ref	0.93 (0.82–1.06)	0.99 (0.87–1.13)	0.89 (0.78–1.02)	0.163
Lutein + zeaxanthin (mcg/kg per day)	ref	0.97 (0.84–1.11)	1.04 (0.91–1.20)	0.98 (0.85–1.14)	0.940
Total carotene intake (mcg/kg per day)	ref	0.99 (0.87–1.13)	0.94 (0.82–1.07)	0.87 (0.76–1.00)	**0.012**

*All analyses adjusted for age, sex, family income-poverty ratio level, race, education level, marital status, alcohol consumption, smoking, and leisure-time physical activity, log-transformed total energy intake, HEI-2015 (if not specified), baseline eGFR, log-transformed urinary ACR, body mass index, total-to-HDL cholesterol ratio, serum phosphorus, hemoglobin, hypertension, and diabetes, and history of cardiovascular disease and cancer.*

*Q, quintile; BMI, body mass index; CVD, cardiovascular disease; CKD, chronic kidney disease; ACR, albumin-Cr ratio.*

*Statistically significant results are shown in bold*.

We conducted subgroup analyses of the associations between total carotene intakes and mortality (Table 4). And the relationship seems to be more obvious and significant in people who were higher educated, fatter, drinkers, and without diabetes than others. In addition, subgroup analyses of the associations between other kinds of carotenoid intakes and mortality also have been conducted (Supplementary Tables 1–5).

**Table 4:** Subgroup analyses of the associations between total carotene intakes and mortality.

**Subgroups**	**Total carotenoids quartile (mcg/kg/d)**				***p* value for interaction**
	**Q1 (≦29.91)**	**Q2 (29.92–69.91)**	**Q3 (69.92–141.89)**	**Q4 (≧141.90)**	
**Age**					0.401
<60 years	ref	0.74 (0.49–1.13)	1.12 (0.76–1.65)	0.87 (0.57–1.35)	
≧60 years	ref	1.02 (0.89–1.16)	0.93 (0.81–1.06)	**0.86 (0.75–0.99)**	
**Sex**					0.877
Male	ref	0.94 (0.79–1.12)	0.93 (0.78–1.11)	0.86 (0.72–1.04)	
Female	ref	1.02 (0.85–1.23)	0.92 (0.76–1.10)	0.84 (0.69–1.02)	
**Race**					0.530
Non-Hispanic white	ref	0.96 (0.82–1.12)	0.90 (0.77–1.06)	**0.80 (0.68–0.94)**	
Others	ref	0.94 (0.77–1.16)	0.88 (0.71–1.10)	0.92 (0.74–1.16)	
**Education levels**					**<0.001**
< High school	ref	1.04 (0.90–1.22)	1.03 (0.88–1.21)	0.95 (0.80–1.12)	
≧High school	ref	0.86 (0.68–1.08)	**0.73 (0.59–0.92)**	**0.66 (0.53–0.83)**	
**Marital status**					0.762
Married	ref	0.97 (0.80–1.18)	0.96 (0.79–1.17)	0.89 (0.72–1.09)	
Unmarried	ref	0.97 (0.83–1.15)	0.89 (0.75–1.04)	**0.81 (0.68–0.96)**	
**BMI**					**0.011**
<25 kg/m^2^	ref	1.07 (0.83–1.37)	0.96 (0.75–1.24)	0.91 (0.72–1.17)	
≧25 kg/m^2^	ref	0.96 (0.83–1.11)	0.92 (0.80–1.07)	**0.84 (0.72–0.99)**	
**Alcohol drinking status**					**0.002**
Abstainer	ref	0.95 (0.78–1.14)	0.96 (0.79–1.16)	0.86 (0.70–1.05)	
Drinker	ref	1.01 (0.85–1.20)	0.90 (0.76–1.07)	**0.83 (0.70–0.98)**	
**Smoking**					0.881
Yes	ref	0.95 (0.80–1.11)	0.92 (0.78–1.09)	**0.83 (0.69–0.99)**	
No	ref	1.06 (0.86–1.29)	0.96 (0.78–1.17)	0.90 (0.73–1.10)	
**Hypertension**					**0.014**
Yes	ref	1.03 (0.89–1.18)	0.94 (0.82–1.08)	0.88 (0.76–1.02)	
No	ref	0.80 (0.59–1.09)	0.90 (0.65–1.25)	0.77 (0.55–1.07)	
**Diabetes**					**0.002**
Yes	ref	1.14 (0.91–1.42)	0.93 (0.73–1.17)	1.00 (0.79–1.28)	
No	ref	0.90 (0.77–1.05)	0.90 (0.77–1.05)	**0.78 (0.67–0.91)**	
**CVD**					0.356
Yes	ref	1.12 (0.93–1.34)	0.87 (0.72–1.05)	0.84 (0.69–1.02)	
No	ref	0.86 (0.72–1.03)	0.98 (0.82–1.16)	0.89 (0.74–1.07)	
**Cancer**					0.628
Yes	ref	1.11 (0.85–1.45)	0.86 (0.66–1.13)	0.88 (0.67–1.15)	
No	ref	0.92 (0.80–1.06)	0.91 (0.79–1.05)	**0.81 (0.70–0.94)**	
**CKD stage**					0.882
1	ref	0.99 (0.66–1.49)	0.82 (0.52–1.27)	0.84 (0.55–1.29)	
2	ref	0.88 (0.67–1.15)	0.98 (0.75–1.27)	0.76 (0.58–1.00)	
3	ref	0.97 (0.82–1.14)	0.87 (0.73–1.03)	0.84 (0.71–1.00)	
4	ref	1.15 (0.78–1.70)	1.19 (0.78–1.83)	1.04 (0.63–1.70)	
**ACR**					0.442
≦30 mg/g	ref	0.95 (0.78–1.17)	0.90 (0.73–1.12)	0.86 (0.69–1.06)	
>30 mg/g	ref	1.01 (0.86–1.19)	0.96 (0.81–1.12)	0.89 (0.75–1.06)	
**Hemoglobin**					
Male: <13.0 g/dL	ref	0.84 (0.62–1.15)	0.86 (0.62–1.18)	**0.60 (0.43–0.86)**	0.081
Male: ≧13.0 g/dL	ref	0.96 (0.78–1.18)	0.97 (0.78–1.20)	0.96 (0.78–1.19)	
Female: <12.0 g/dL	ref	1.07 (0.70–1.62)	0.91 (0.59–1.40)	0.81 (0.49–1.33)	**0.001**
Female: ≧12.0 g/dL	ref	1.00 (0.81–1.24)	0.91 (0.74–1.13)	0.85 (0.68–1.06)	
**Serum phosphorus**					0.901
≦3.05 mg/dL	ref	0.86 (0.57–1.32)	1.02 (0.65–1.60)	0.78 (0.48–1.26)	
	**Q1 (≦29.91)**	**Q2 (29.92–69.91)**	**Q3 (69.92–141.89)**	**Q4 (≧141.90)**	
3.06–4.45 mg/dL	ref	0.95 (0.83–1.10)	0.88 (0.76–1.01)	**0.82 (0.71–0.95)**	
≦4.46 mg/dL	ref	1.24 (0.83–1.84)	1.18 (0.78–1.80)	1.16 (0.74–1.82)	

*Adjusted covariates: age, sex, family income-poverty ratio level, race, education level, marital status, alcohol consumption, smoking, and leisure-time physical activity, log-transformed total energy intake, HEI-2015, baseline eGFR, log-transformed urinary ACR, body mass index, total-to-HDL cholesterol ratio, serum phosphorus, hemoglobin, hypertension, and diabetes, and history of cardiovascular disease and cancer. The variable used for stratification was not included in the given model. Interaction was tested using a continuous total carotene intake term and the exposure of interest, using a Wald test for dichotomous variables.*

*Q, quintile; BMI; body mass index; CVD, cardiovascular disease; CKD, chronic kidney disease; ACR, albumin-Cr ratio.*

*Statistically significant results are shown in bold*.

Table 5 shows the associations of the quartiles of the serum carotenoid levels relative to Quartile 1 with mortality among the total participants both in the unadjusted and adjusted model. In addition to the α-carotene, the rest of the four baseline serum carotenoid levels were associated with the decline of death in CKD patients. When adjustments were made for model 1, the risk of death was reduced in CKD participants with these five higher serum carotenoids levels (*P* < 0.05). And this result still persisted in α-carotene (HR = 0.77, 95%CI, 0.65-0.92, *P* = 0.002), β-cryptoxanthin (HR = 0.83, 95%CI, 0.70–0.98, *P* = 0.019), Lycopene (HR = 0.77, 95%CI, 0.65–0.91, *P* = 0.002), and Lutein + zeaxanthin (HR = 0.82, 95%CI, 0.70–0.96, *P* = 0.002) after adjusting for Model 2. However, it becomes less pronounced in Model 2 of serum β-carotene (HR = 0.80, 95%CI, 0.67–0.95, *P* = 0.079). Results were not substantially changed when we further adjusted for substituting HEI-2015 by food groups, substituting HEI-2015 by macronutrients, waist circumference instead of BMI, or excluding those who died within 1 year (Table 6). The dose-response analysis of the associations of serum carotene with mortality shows the same result and finds that a non-linear relationship was not indicated in β-carotene, β-cryptoxanthin, and lycopene (Supplementary Figure 2). And it's worth mentioning that the result of serum β-cryptoxanthin (HR = 0.83, 95%CI, 0.70-0.98, *P* = 0.019) was completely different from that of dietary (HR = 1.15, 95%CI, 1.00–1.31, *P* = 0.013) In addition, the dietary intake of α-carotene, β-carotene, β-cryptoxanthin, lycopene, as well as lutein + zeaxanthin were weakly linear correlated to the correspondence serum contents (Supplementary Table 6). Subgroup analyses of the associations between serum carotenoid levels and mortality have been conducted (Supplementary Tables 7–11).

**Table 5:** The associations of the quartile of serum carotenoids, relative to Quartile 1 with mortality.

	**Cutoff**	**Cases/participants**	**Incidence Rate per 1,000 Person-Years (95% CI)**	**Crude**	**Model 1**	**Model 2**
				**HR (95% CI)**	**HR (95% CI)**	**HR (95% CI)**
**Alpha-carotene (ug/dL)**						
Quartile 1	≦1.50	311/633	55.7 (49.9–62.1)	ref	ref	ref
Quartile 2	1.51–2.80	288/594	54.9 (49.1–61.5)	0.99 (0.84–1.16)	0.86 (0.73–1.01)	0.85 (0.72–1.01)
Quartile 3	2.81–5.20	307/613	55.5 (49.7–61.9)	1.00 (0.85–1.17)	**0.75 (0.64–0.89)**	**0.79 (0.67–0.93)**
Quartile 4	At least 5.21	295/605	53.6 (47.9–60.0)	0.96 (0.82–1.13)	**0.71 (0.60–0.85)**	**0.77 (0.65–0.92)**
P trend				0.689	**<0.001**	**0.002**
**Beta-carotene (ug/dL)**						
Quartile 1	≦8.49	264/599	47.7 (42.3–53.7)	ref	ref	ref
Quartile 2	8.50–15.29	281/592	52.9 (47.1–59.4)	1.12 (0.94–1.32)	**0.78 (0.65–0.92)**	**0.74 (0.62–0.88)**
Quartile 3	15.30–27.02	294/593	55.2 (49.3–61.8)	1.16 (0.98–1.37)	**0.78 (0.65–0.93)**	**0.78 (0.66–0.94)**
Quartile 4	At least 27.03	337/593	**65.4 (58.9–72.6)**	**1.39 (1.18–1.63)**	**0.76 (0.64–0.91)**	**0.80 (0.67–0.95)**
P trend				**<0.001**	**0.012**	0.079
**Beta-cryptoxanthin (ug/dL)**						
Quartile 1	≦5.00	354/645	65.7 (59.3–72.7)	ref	ref	ref
Quartile 2	5.01–8.62	295/623	**52.6 (47.0–58.8)**	**0.79 (0.68–0.93)**	0.92 (0.79–1.08)	0.93 (0.79–1.08)
Quartile 3	8.63–13.44	290/587	**55.0 (49.1–61.6)**	**0.83 (0.71–0.97)**	**0.83 (0.71–0.97)**	0.86 (0.74–1.01)
Quartile 4	At least 13.45	262/584	**47.3 (41.9–53.3)**	**0.71 (0.61–0.83)**	**0.76 (0.65–0.91)**	**0.83 (0.70–0.98)**
P trend				**<0.001**	**0.001**	**0.019**
**Lycopene (ug/dL)**						
Quartile 1	≦14.90	409/612	82.5 (75.1–90.6)	ref	ref	ref
Quartile 2	14.91–25.50	317/610	**56.4 (50.6–62.8)**	**0.67 (0.58–0.78)**	**0.80 (0.69–0.93)**	**0.81 (0.70–0.94)**
Quartile 3	25.51–38.64	255/607	**45.2 (40.4–51.0)**	**0.54 (0.46–0.63)**	**0.74 (0.63–0.87)**	**0.79 (0.67–0.92)**
Quartile 4	At least 38.65	221/609	39.6 (34.7–45.1)	**0.47 (0.40–0.55)**	**0.76 (0.64–0.89)**	**0.77 (0.65–0.91)**
P trend				**<0.001**	**<0.001**	**0.002**
**Lutein** **+** **zeaxanthin (ug/dL)**						
Quartile 1	≦10.40	337/613	64.7 (58.2–71.8)	ref	ref	ref
Quartile 2	10.41–14.72	301/610	**55.2 (49.4–61.7)**	**0.85 (0.73–0.99)**	**0.77 (0.65–0.90)**	**0.82 (0.70–0.96)**
Quartile 3	14.73–20.80	261/617	**45.0 (39.9–50.7)**	**0.69 (0.59–0.81)**	**0.65 (0.55–0.77)**	**0.68 (0.58–0.81)**
Quartile 4	At least 20.81	303/605	**56.2 (50.3–62.8)**	0.86 (0.74–1.01)	**0.74 (0.63–0.87)**	**0.82 (0.70–0.96)**
P trend				**0.01**	**<0.001**	**0.002**

*Model 1 adjusted for age, sex, family income-poverty ratio level, race, education level, marital status, alcohol consumption, smoking, and leisure-time physical activity.*

*Model 2 additionally adjusted for log-transformed total energy intake, HEI-2015, baseline eGFR, log-transformed urinary ACR, body mass index, total-to-HDL cholesterol ratio, serum phosphorus, hemoglobin, hypertension, and diabetes, and history of cardiovascular disease and cancer.*

*HR, hazard ratio; CI, confidence interval; HDL, high density lipoprotein; HEI-2015, healthy eating index score-2015; eGFR, estimated glomerular filtration rate; ACR, albumin-Cr ratio.*

*Statistically significant results are shown in bold*.

**Table 6:** Results of sensitivity analyses of the associations between serum carotenoids and mortality.

	**Q1**	**Q2**	**Q3**	**Q4**	**P trend**
**Substituting HEI-2015 by food groups**				
Alpha-carotene (ug/dL)	ref	0.87 (0.74-1.02)	**0.80 (0.68-0.94)**	**0.78 (0.65-0.93)**	**0.003**
Beta-carotene (ug/dL)	ref	**0.76 (0.64-0.90)**	**0.80 (0.67-0.96)**	**0.82 (0.68-0.98)**	0.123
Beta-cryptoxanthin (ug/dL)	ref	0.93 (0.79-1.08)	0.85 (0.72-1.00)	**0.80 (0.67-0.96)**	**0.008**
Lycopene (ug/dL)	ref	**0.80 (0.69-0.93)**	**0.79 (0.68-0.93)**	**0.78 (0.66-0.92)**	**0.003**
Lutein + zeaxanthin (ug/dL)	ref	**0.83 (0.71-0.97)**	**0.68 (0.58-0.80)**	**0.81 (0.69-0.95)**	**0.001**
**Substituting HEI-2015 by macronutrients**				
Alpha-carotene (ug/dL)	ref	0.87 (0.74-1.02)	**0.80 (0.68-0.94)**	**0.78 (0.65-0.93)**	**0.003**
Beta-carotene (ug/dL)	ref	**0.76 (0.64-0.90)**	**0.80 (0.67-0.96)**	**0.82 (0.68-0.98)**	0.123
Beta-cryptoxanthin (ug/dL)	ref	0.94 (0.80-1.09)	0.86 (0.74-1.01)	**0.83 (0.7-0.98)**	**0.017**
Lycopene (ug/dL)	ref	**0.80 (0.69-0.93)**	**0.79 (0.68-0.93)**	**0.78 (0.66-0.92)**	**0.003**
Lutein + zeaxanthin (ug/dL)	ref	**0.83 (0.71-0.97)**	**0.68 (0.58-0.80)**	**0.81 (0.69-0.95)**	**0.001**
**Adjusting for waist circumference instead of BMI**			
Alpha-carotene (ug/dL)	ref	0.87 (0.74-1.03)	**0.80 (0.68-0.94)**	**0.77 (0.65-0.92)**	**0.002**
Beta-carotene (ug/dL)	ref	**0.75 (0.63-0.90)**	**0.81 (0.68-0.97)**	**0.83 (0.69-0.99)**	0.180
Beta-cryptoxanthin (ug/dL)	ref	0.94 (0.80-1.09)	0.85 (0.73-1.00)	**0.83 (0.70-0.98)**	**0.012**
Lycopene (ug/dL)	ref	**0.79 (0.68-0.91)**	**0.78 (0.67-0.92)**	**0.76 (0.65-0.90)**	**0.001**
Lutein + zeaxanthin (ug/dL)	ref	**0.83 (0.71-0.97)**	**0.68 (0.58-0.80)**	**0.82 (0.70-0.96)**	**0.001**
**Excluding those died within 1 year**				
Alpha-carotene (ug/dL)	ref	0.89 (0.75-1.06)	0.84 (0.71-1.00)	**0.78 (0.65-0.94)**	**0.007**
Beta-carotene (ug/dL)	ref	**0.77 (0.64-0.92)**	**0.80 (0.67-0.96)**	0.83 (0.69-1.00)	0.181
Beta-cryptoxanthin (ug/dL)	ref	0.94 (0.80-1.1.00)	0.87 (0.74-1.03)	0.85 (0.71-1.01)	**0.043**
Lycopene (ug/dL)	ref	**0.81 (0.69-0.94)**	**0.78 (0.66-0.92)**	**0.78 (0.65-0.93)**	**0.002**
Lutein + zeaxanthin (ug/dL)	ref	0.85 (0.72-1.00)	**0.71 (0.60-0.84)**	**0.81 (0.68-0.95)**	**0.002**

*All analyses adjusted for age, sex, family income-poverty ratio level, race, education level, marital status, alcohol consumption, smoking, and leisure-time physical activity, log-transformed total energy intake, HEI-2015 (if not specified), baseline eGFR, log-transformed urinary ACR, body mass index, total-to-HDL cholesterol ratio, serum phosphorus, hemoglobin, hypertension, and diabetes, and history of cardiovascular disease and cancer.*

*Q, quintile; BMI, body mass index; CVD, cardiovascular disease; CKD, chronic kidney disease; ACR, albumin-Cr ratio.*

*Statistically significant results are shown in bold*.

## Discussion

In this population-based retrospective cohort study, higher intake of total carotene was inversely associated with the mortality of CKD patients after adjustment for potential confounders. And the serum concentration of carotenoid is also showing that the high level of α-carotene, β-cryptoxanthin, lycopene, as well as lutein + zeaxanthin was significantly associated with decreased all-cause mortality in CKD patients. The dietary modification to increase carotenoid intake represents a possible route for the amelioration prognosis of CKD patients.

Our research substantiated that a higher intake of total carotenoids was inversely associated with mortality of CKD patients after adjustment for potential confounders. We consider that more carotenoids can be intake by CKD patients to improve the prognosis of the disease. Although the mechanism is not clear yet, considerable research has indicated that carotenoids may improve the prognosis of CKD patients through its antioxidant properties (9). Oxidative stress is a contributor to many diseases, and shown kidney disease is associated with permanent inflammation accompanied by oxidative stress (9). The disturbance of the redox balance is associated with an increase in ROS production and a decrease in antioxidant capacity. As the CKD progresses, a gradual elevation of oxidative damage with a reduction in endogenous antioxidant defenses (35). Furthermore, over-production of the ROS may lead to renal fibrosis, inflammation, and endothelial dysfunction (12, 36). These are known risk factors for the onset of serious systemic complications, cardiovascular disease, anemia, and mineral disorders, which are closely related to death in CKD patients. Therefore, carotenoids, which beneficial effects are mainly derived from their antioxidant properties as the main scavenger of ROS such as singlet molecular oxygen (O_2_), can indeed improve the prognosis to some extent. However, each carotenoid has its unique antioxidant properties, and which kind influences the mortality of CKD most has not been clear yet.

Moreover, both the cox regression analysis and dose-response analysis recognized that the β-carotene and lycopene intake do reduce the mortality of CKD patients while other carotenoids do not. These differences may be due to the following reasons. First, different carotenoids have different antioxidant activities according to their structure and environmental polarity. For instance, the efficiency scavengers of free radicals are different in polar carotenoids (lutein + zeaxanthin and β-cryptoxanthin) and non-polar hydrocarbon carotenoids (α-carotene, β-carotene, and lycopene) (12). Each carotenoid's antioxidant properties depend on its unique functional group and magnitude of conjugated double-bonds (12). Secondly, the potential beneficial effects of a carotenoid may depend on the concentrations of another (14). Different carotenoids interact with each other, contributing to the different impacts on CKD patients. Lastly, partial carotenoids can act as prooxidants at high carotenoid concentrations or high oxygen concentrations (12, 37). Indeed, this property was proposed by Burton as early as 1984 that when under a higher partial pressure of oxygen and higher concentration of carotenoids (> 500 μM), there will be a transition from antioxidant behavior to pro-oxidative behavior (38). And the subsequent experiments *in vivo* and *in vitro* also confirmed this. All in all, the study on the antioxidant activity of carotenoids was not complete yet (37, 38). And it seems to be more beneficial to intake β-carotene and lycopene rather than other carotenoids for patients with CKD. However, results were substantially changed when we further adjusted for substituting waist circumference instead of BMI or excluding those who died within 1 year, while high-level of β-carotene and lycopene intake was not correlated with the decreased mortality of CKD patients. More research into the effects of β-carotene and lycopene intake is required.

In addition, we found that the carotenoid may be more useful in specific groups of people such as the high-educated, non-Hispanic white, fatter, drinker, and without diabetes through the subgroup analysis of total carotenoid intake. This might partly be due to the difference in the CKD population, which has been verified that can affect the prognosis of patients yet, such as diabetes and obesity. Furthermore, the patients' redox status can be affected by many factors like CKD stage and age, which are attributed to the metabolism status of carotenoids in the body (39). And the bioavailability and absorption of each carotenoid may be effect by multi-factors, such as the carotenoid type, isomeric forms, interaction with fat and fiber, aging, and nutritional status (40). Finally, the different genetic backgrounds and lifestyles between non-Hispanic whites and others may attribute to the diversity. And despite that, the causes of these factors' associations with carotenoid intake effect in CKD patients are not yet fully understood. This conclusion provides more reference for the choice of clinicians. And more prospective studies and fundamental research should be done nearly.

Interestingly, the results between dietary intake and serum concentration of other carotenoids are not exactly coincidental though most of the serum carotenoids show a negative correlation with the mortality of CKD patients. This may be caused by two reasons. On one hand, the bioavailability of each carotenoid is not consistent at all. And the absorption, distribution, and metabolism of every kind of carotenoid are not exactly consistent, too. The increase of oral carotenoids is not necessarily followed by an increase in carotenoid concentration. On the other hand, the limitations in estimation methods of the oral carotenoids contribute to the instability of our results. These differences remind us that we should put more attention to the sub carotenoid research, and the improvements in methodology are crucial. In any case, the high-level of total carotenoid dietary intake does negatively correlated with the all-cause mortality of CKD patients, and more reliable prospective research should be conducted.

To the best of our knowledge, this research was the first retrospective cohort study that assessed the relation of dietary intake of carotene and serum carotene with the mortality of CKD patients of the NHANES database. And our study had several strengths including the large sample size and the well-characterized study population enabling appropriate adjustment for confounding effects. We conducted several analyses such as dose-response analysis and sensitivity analyses to demonstrate our results to ensure reliability.

Some important limitations deserve mention. First, our retrospective study design has inherent limitations consistent with that of other retrospective studies. Second, several variables were dependent on self-reported data and may be subject to recall bias. Third, although we have adjusted for lots of confounding factors, there may still be some unrecognized confounding factors which we couldn't control. Finally, the study population is limited to US adults, which makes it difficult to generalized our findings to the broader community of patients with CKD.

## Conclusions

Overall, in this retrospective cohort study of the US CKD population, we found that higher intake of total carotene was significantly associated with a lower risk of death. In addition to β-carotene, other kinds of serum carotenoids were also significantly associated with the mortality of CKD patients. These findings are promising given the further therapeutic options for treating CKD.

## Data Availability Statement

The datasets presented in this study can be found in online repositories. The names of the repository/repositories and accession number(s) can be found below: https://www.cdc.gov/nchs/nhanes/participant.htm.

## Ethics Statement

The studies involving human participants were reviewed and approved by NCHS Research Ethics Review Board. The patients/participants provided their written informed consent to participate in this study.

## Author Contributions

Conceptualization and data curation: YH and XC. Methodology: XC and HG. Project administration: NZ, SG, and YY. Software: NZ. Supervision and writing—review and editing: SG and YY. Validation: YL and YM. Writing—original draft: YH and HG. All authors contributed to the article and approved the submitted version.

## Conflict of Interest

The authors declare that the research was conducted in the absence of any commercial or financial relationships that could be construed as a potential conflict of interest.

## Publisher's Note

All claims expressed in this article are solely those of the authors and do not necessarily represent those of their affiliated organizations, or those of the publisher, the editors and the reviewers. Any product that may be evaluated in this article, or claim that may be made by its manufacturer, is not guaranteed or endorsed by the publisher.
