# Supplementary material for: Relation Between Dietary Carotenoid Intake, Serum Concentration, and Mortality Risk of CKD Patients Among US Adults: National Health and Nutrition Examination Survey 2001–2014
Source: Front Med (Lausanne). 2022 Jul 8;9:871767. doi: 10.3389/fmed.2022.871767 (PMC9304649; doi:10.3389/fmed.2022.871767)
Supplement: Supplementary file 1 [file Data_Sheet_1.docx]

**Supplementary Table 1** Subgroup analyses of the associations between alpha-carotene intake and mortality.

| subgroups | Alpha-carotene intake (mcg/kg per day) | | | | p value for interaction |
| --- | --- | --- | --- | --- | --- |
|  | Q1 (≦0.29) | Q2 (0.30-1.05) | Q3 (1.06-5.53) | Q4 (≧5.54) |  |
| Age |  |  |  |  | **0.041** |
| ﹤60 years | ref | 1.30 (0.88-1.91) | 1.19 (0.78-1.80) | 1.06 (0.67-1.67) |  |
| ≧60 years | ref | 0.89 (0.77-1.01) | **0.87 (0.76-0.99)** | 0.95 (0.83-1.08) |  |
| Sex |  |  |  |  | 0.791 |
| Male | ref | 0.97 (0.82-1.16) | 0.88 (0.73-1.05) | 0.90 (0.75-1.08) |  |
| Female | ref | 0.84 (0.69-1.02) | 0.86 (0.71-1.04) | 0.99 (0.82-1.20) |  |
| Race |  |  |  |  | **<0.001** |
| Non-Hispanic white | ref | 0.92 (0.78-1.09) | 0.90 (0.76-1.06) | 1.05 (0.89-1.24) |  |
| Others | ref | 0.92 (0.75-1.13) | 0.85 (0.68-1.05) | **0.77 (0.62-0.97)** |  |
| Education levels |  |  |  |  | 0.282 |
| ﹤High school | ref | 0.94 (0.81-1.11) | 0.90 (0.77-1.06) | 1.00 (0.85-1.18) |  |
| ≧High school | ref | 0.83 (0.66-1.05) | 0.81 (0.64-1.03) | 0.88 (0.69-1.12) |  |
| Marital status |  |  |  |  | 0.552 |
| Married | ref | 0.99 (0.81-1.21) | 0.93 (0.76-1.14) | 0.95 (0.77-1.17) |  |
| Unmarried | ref | 0.86 (0.73-1.02) | 0.85 (0.72-1.00) | 0.93 (0.78-1.10) |  |
| BMI |  |  |  |  | 0.369 |
| ﹤25 kg/m^2^ | ref | 1.09 (0.84-1.42) | 0.98 (0.76-1.27) | 0.90 (0.70-1.16) |  |
| ≧25 kg/m^2^ | ref | 0.97 (0.83-1.12) | 0.91 (0.78-1.06) | 0.94 (0.80-1.11) |  |
| Alcohol drinking status |  |  |  |  | 0.385 |
| Abstainer | ref | 0.93 (0.76-1.13) | 0.91 (0.75-1.11) | 0.97 (0.79-1.19) |  |
| Drinker | ref | 0.89 (0.75-1.06) | 0.85 (0.71-1.02) | 0.94 (0.78-1.12) |  |
| Smoking |  |  |  |  | 0.469 |
| Yes | ref | 0.89 (0.75-1.05) | 0.90 (0.76-1.07) | 0.91 (0.76-1.09) |  |
| No | ref | 0.93 (0.76-1.15) | 0.84 (0.68-1.04) | 0.99 (0.81-1.22) |  |
| Hypertension |  |  |  |  | **0.026** |
| Yes | ref | 0.93 (0.80-1.07) | 0.91 (0.79-1.05) | 0.90 (0.78-1.04) |  |
| No | ref | 0.78 (0.57-1.07) | 0.76 (0.55-1.04) | 0.86 (0.63-1.19) |  |
| Diabetes |  |  |  |  | 0.865 |
| Yes | ref | 0.97 (0.77-1.22) | 0.98 (0.78-1.25) | 0.97 (0.75-1.25) |  |
| No | ref | 0.88 (0.75-1.02) | **0.81 (0.69-0.95)** | 0.92 (0.79-1.07) |  |
| CVD |  |  |  |  | 0.582 |
| Yes | ref | 0.93 (0.77-1.12) | 0.94 (0.77-1.14) | 0.95 (0.77-1.17) |  |
| No | ref | 0.91 (0.76-1.08) | 0.84 (0.70-1.01) | 0.98 (0.82-1.18) |  |
| Cancer |  |  |  |  | **0.031** |
| Yes | ref | 0.77 (0.59-1.00) | **0.70 (0.54-0.91)** | 0.93 (0.72-1.21) |  |
| No | ref | 0.95 (0.82-1.10) | 0.92 (0.79-1.07) | 0.93 (0.80-1.09) |  |
| CKD stage |  |  |  |  | 0.271 |
| 1 | ref | 0.99 (0.65-1.49) | 1.05 (0.68-1.62) | 1.24 (0.80-1.92) |  |
| 2 | ref | 0.95 (0.72-1.24) | 0.88 (0.66-1.17) | 0.88 (0.66-1.18) |  |
| 3 | ref | 0.89 (0.75-1.07) | 0.88 (0.74-1.05) | 0.97 (0.81-1.17) |  |
| 4 | ref | 0.84 (0.55-1.28) | 0.74 (0.48-1.13) | 0.87 (0.57-1.32) |  |
| ACR |  |  |  |  | 0.366 |
| ≦30mg/g | ref | **0.76 (0.61-0.94)** | 0.82 (0.67-1.00) | 0.88 (0.72-1.08) |  |
| ﹥30mg/g | ref | 0.98 (0.83-1.15) | 0.89 (0.75-1.06) | 1.01 (0.84-1.20) |  |
| Hemoglobin |  |  |  |  |  |
| Male: ﹤13.0 g/dL | ref | 1.07 (0.77-1.47) | 0.74 (0.54-1.03) | 0.82 (0.59-1.13) | **0.035** |
| Male: ≧13.0 g/dL | ref | 0.98 (0.80-1.21) | 0.94 (0.75-1.17) | 0.95 (0.76-1.19) |  |
| Female: ﹤12.0 g/dL | ref | 0.89 (0.58-1.35) | 0.78 (0.51-1.20) | 1.01 (0.65-1.56) | 0.967 |
| Female: ≧12.0 g/dL | ref | 0.82 (0.66-1.03) | 0.90 (0.73-1.12) | 1.01 (0.82-1.26) |  |
| Serum phosphorus |  |  |  |  | 0.284 |
| ≦3.05 mg/dL | ref | 0.82 (0.53-1.28) | 0.73 (0.47-1.13) | 0.92 (0.60-1.41) |  |
| 3.06-4.45 mg/dL | ref | 0.93 (0.80-1.08) | 0.91 (0.79-1.06) | 0.95 (0.81-1.10) |  |
| ﹥4.46 mg/dL | ref | 0.82 (0.55-1.23) | 0.72 (0.48-1.08) | 1.06 (0.71-1.57) |  |

Adjusted covariates: age, sex, family income-poverty ratio level, race, education level, marital status, alcohol consumption, smoking, and leisure-time physical activity, log-transformed total energy intake, HEI-2015, baseline eGFR, log-transformed urinary ACR, body mass index, total-to-HDL cholesterol ratio, serum phosphorus, hemoglobin, hypertension, and diabetes, and history of cardiovascular disease and cancer. The variable used for stratification was not included in the given model. Interaction was tested using a continuous total carotene intake term and the exposure of interest, using a Wald test for dichotomous variables.

*Q* quintile, BMI body mass index, CVD cardiovascular disease, CKD chronic kidney disease, ACR albumin-Cr ratio.

**Supplementary Table 2** Subgroup analyses of the associations between beta-carotene intakes and mortality.

| subgroups | Beta-carotene intake (mcg/kg per day) | | | | | p value for interaction |
| --- | --- | --- | --- | --- | --- | --- |
|  | Q1 (≦4.90) | Q2 (4.91-13.16) | | Q3 (13.17-34.21) | Q4 (≧34.22) |  |
| Age |  |  | |  |  | 0.041 |
| ﹤60 years | ref | 0.77 (0.52-1.13) | | 1.03 (0.68-1.54) | 1.08 (0.70-1.66) |  |
| ≧60 years | ref | 0.92 (0.80-1.06) | | 0.92 (0.80-1.06) | 0.92 (0.80-1.05) |  |
| Sex |  |  | |  |  | 0.916 |
| Male | ref | 0.99 (0.83-1.18) | | 0.93 (0.78-1.11) | 0.90 (0.74-1.08) |  |
| Female | ref | **0.77 (0.64-0.94)** | | **0.81 (0.67-0.98)** | **0.81 (0.68-0.98)** |  |
| Race |  |  | |  |  | 0.476 |
| Non-Hispanic white | ref | 0.95 (0.80-1.12) | | 0.94 (0.80-1.12) | 0.97 (0.81-1.15) |  |
| Others | ref | 0.81 (0.66-1.01) | | 0.81 (0.65-1.00) | **0.75 (0.61-0.93)** |  |
| Education levels |  |  | |  |  | 0.078 |
| ﹤High school | ref | 0.86 (0.74-1.01) | | 0.90 (0.77-1.05) | **0.85 (0.73-0.99)** |  |
| ≧High school | ref | 0.91 (0.71-1.15) | | 0.82 (0.64-1.04) | 0.84 (0.65-1.07) |  |
| Marital status |  |  | |  |  | **0.004** |
| Married | ref | 0.94 (0.76-1.15) | | 1.00 (0.82-1.23) | 0.97 (0.78-1.21) |  |
| Unmarried | ref | 0.89 (0.75-1.06) | | **0.80 (0.67-0.95)** | **0.80 (0.68-0.94)** |  |
| BMI |  |  | |  |  | 0.755 |
| ﹤25 kg/m^2^ | ref | 1.09 (0.84-1.42) | | 0.98 (0.76-1.27) | 0.90 (0.70-1.16) |  |
| ≧25 kg/m^2^ | ref | **0.85 (0.74-0.99)** | | 0.86 (0.74-1.00) | 0.89 (0.76-1.03) |  |
| Alcohol drinking status |  |  | |  |  | 0.612 |
| Abstainer | ref | **0.80 (0.66-0.96)** | | **0.78 (0.64-0.94)** | **0.81 (0.67-0.98)** |  |
| Drinker | ref | 0.97 (0.82-1.16) | | 0.96 (0.80-1.14) | 0.88 (0.74-1.06) |  |
| Smoking |  |  | |  |  | **0.036** |
| Yes | ref | 1.03 (0.87-1.21) | | 0.99 (0.83-1.17) | 0.86 (0.72-1.04) |  |
| No | ref | **0.71 (0.58-0.87)** | | **0.73 (0.60-0.90)** | 0.82 (0.68-1.00) |  |
| Hypertension |  |  | |  |  | **0.019** |
| Yes | ref | 0.93 (0.80-1.07) | | 0.91 (0.79-1.05) | 0.90 (0.78-1.04) |  |
| No | ref | 0.78 (0.57-1.07) | | 0.79 (0.57-1.10) | 0.77 (0.55-1.06) |  |
| Diabetes |  |  | |  |  | 0.667 |
| Yes | ref | 1.13 (0.91-1.40) | | 0.82 (0.65-1.03) | 0.87 (0.69-1.10) |  |
| No | ref | **0.78 (0.66-0.91)** | | 0.87 (0.74-1.01) | **0.82 (0.70-0.95)** |  |
| CVD |  |  | |  |  | **0.043** |
| Yes | ref | 1.00 (0.83-1.21) | | 0.92 (0.76-1.12) | 0.86 (0.70-1.05) |  |
| No | ref | **0.81 (0.68-0.97)** | | 0.84 (0.70-1.01) | 0.91 (0.76-1.09) |  |
| Cancer |  |  | |  |  | **0.006** |
| Yes | ref | 0.96 (0.72-1.28) | | 0.87 (0.66-1.15) | 1.02 (0.77-1.36) |  |
| No | ref | 0.87 (0.75-1.01) | | **0.85 (0.74-0.99)** | **0.79 (0.68-0.92)** |  |
| CKD stage |  |  | |  |  | **0.014** |
| 1 | ref | 1.12 (0.73-1.70) | | 1.09 (0.71-1.68) | 1.04 (0.66-1.63) |  |
| 2 | ref | 1.00 (0.76-1.32) | | 0.91 (0.68-1.23) | 0.95 (0.70-1.29) |  |
| 3 | ref | **0.80 (0.67-0.96)** | | 0.84 (0.71-1.00) | **0.82 (0.69-0.97)** |  |
| 4 | ref | 0.90 (0.60-1.36) | | 0.85 (0.56-1.30) | 0.80 (0.49-1.29) |  |
| ACR |  |  | |  |  | 0.786 |
| ≦30mg/g | ref | 0.88 (0.71-1.10) | | 0.92 (0.74-1.14) | 0.87 (0.70-1.08) |  |
| ﹥30mg/g | ref | 0.93 (0.79-1.09) | | 0.90 (0.76-1.06) | 0.92 (0.77-1.10) |  |
| Hemoglobin |  | |  |  |  |  |
| Male: ﹤13.0 g/dL | ref | | 1.04 (0.76-1.42) | 0.72 (0.52-1.01) | 0.71 (0.51-1.00) | 0.677 |
| Male: ≧13.0 g/dL | ref | | 0.99 (0.80-1.22) | 1.02 (0.82-1.26) | 0.96 (0.76-1.20) |  |
| Female: ﹤12.0 g/dL | ref | | **0.62 (0.40-0.96)** | **0.65 (0.42-0.99)** | 0.75 (0.49-1.13) | **0.043** |
| Female: ≧12.0 g/dL | ref | | 0.88 (0.70-1.10) | 0.91 (0.72-1.13) | 0.91 (0.72-1.14) |  |
| Serum phosphorus |  | |  |  |  | **0.004** |
| ≦3.05 mg/dL | ref | | 0.94 (0.60-1.47) | 0.72 (0.48-1.09) | 0.91 (0.59-1.41) |  |
| 3.06-4.45 mg/dL | ref | | 0.87 (0.76-1.01) | 0.88 (0.76-1.02) | **0.82 (0.71-0.95)** |  |
| ﹥4.46 mg/dL | ref | | 1.08 (0.71-1.65) | 0.93 (0.60-1.42) | 1.02 (0.66-1.57) |  |

Adjusted covariates: age, sex, family income-poverty ratio level, race, education level, marital status, alcohol consumption, smoking, and leisure-time physical activity, log-transformed total energy intake, HEI-2015, baseline eGFR, log-transformed urinary ACR, body mass index, total-to-HDL cholesterol ratio, serum phosphorus, hemoglobin, hypertension, and diabetes, and history of cardiovascular disease and cancer. The variable used for stratification was not included in the given model. Interaction was tested using a continuous total carotene intake term and the exposure of interest, using a Wald test for dichotomous variables.

*Q* quintile, BMI body mass index, CVD cardiovascular disease, CKD chronic kidney disease, ACR albumin-Cr ratio.

**Supplementary Table 3** Subgroup analyses of the associations between beta-cryptoxanthin intake and mortality.

| subgroups | | Beta-cryptoxanthin intake (mcg/kg per day) | | | | p value for interaction |
| --- | --- | --- | --- | --- | --- | --- |
|  |  | Q1 (≦0.15) | Q2 (0.16-0.60) | Q3 (0.61-1.77) | Q4 (≧1.78) |  |
| Age | |  |  |  |  | **0.008** |
| ﹤60 years | | ref | 1.27 (0.86-1.89) | 1.36 (0.88-2.09) | 1.24 (0.81-1.90) |  |
| ≧60 years | | ref | 0.96 (0.83-1.11) | **1.17 (1.02-1.35)** | **1.19 (1.04-1.36)** |  |
| Sex | |  |  |  |  | 0.826 |
| Male | | ref | 0.99 (0.83-1.18) | 0.93 (0.78-1.11) | 0.90 (0.74-1.08) |  |
| Female | | ref | 0.94 (0.76-1.15) | 1.14 (0.94-1.39) | 1.04 (0.86-1.25) |  |
| Race | |  |  |  |  | **<0.001** |
| Non-Hispanic white | | ref | 0.96 (0.80-1.14) | 1.17 (0.99-1.39) | 1.17 (0.99-1.38) |  |
| Others | | ref | 1.07 (0.86-1.34) | 1.11 (0.88-1.39) | 1.13 (0.90-1.41) |  |
| Education levels | |  |  |  |  | 0.451 |
| ﹤High school | | ref | 1.06 (0.89-1.25) | 1.24 (1.05-1.46) | **1.21 (1.02-1.42)** |  |
| ≧High school | | ref | 0.86 (0.67-1.09) | 0.99 (0.78-1.25) | 1.00 (0.80-1.26) |  |
| Marital status | |  |  |  |  | 0.292 |
| Married | | ref | 0.92 (0.75-1.14) | 1.18 (0.96-1.45) | **1.25 (1.02-1.53)** |  |
| Unmarried | | ref | 1.05 (0.87-1.26) | 1.13 (0.94-1.36) | 1.10 (0.92-1.31) |  |
| BMI | |  |  |  |  | 0.812 |
| ﹤25 kg/m^2^ | | ref | 0.91 (0.70-1.18) | 1.10 (0.85-1.40) | 1.04 (0.81-1.33) |  |
| ≧25 kg/m^2^ | | ref | 1.04 (0.89-1.23) | **1.18 (1.00-1.39)** | **1.19 (1.02-1.39)** |  |
| Alcohol drinking status | |  |  |  |  | **0.029** |
| Abstainer | | ref | 0.92 (0.75-1.13) | 1.16 (0.95-1.42) | 1.17 (0.96-1.42) |  |
| Drinker | | ref | 1.04 (0.86-1.25) | 1.11 (0.92-1.33) | 1.08 (0.91-1.30) |  |
| Smoking | |  |  |  |  | **0.032** |
| Yes | | ref | 0.94 (0.79-1.11) | 1.07 (0.90-1.28) | 1.08 (0.91-1.28) |  |
| No | | ref | 1.16 (0.92-1.45) | **1.28 (1.04-1.58)** | **1.25 (1.02-1.53)** |  |
| Hypertension | |  |  |  |  | 0.791 |
| Yes | | ref | 0.95 (0.82-1.11) | 1.13 (0.98-1.31) | 1.12 (0.97-1.29) |  |
| No | | ref | 1.26 (0.90-1.78) | 1.39 (0.99-1.96) | 1.29 (0.94-1.78) |  |
| Diabetes | |  |  |  |  | **<0.001** |
| Yes | | ref | 1.04 (0.81-1.32) | **1.31 (1.04-1.66)** | **1.29 (1.02-1.64)** |  |
| No | | ref | 0.96 (0.81-1.14) | 1.08 (0.91-1.27) | 1.07 (0.91-1.25) |  |
| CVD | |  |  |  |  | 0.505 |
| Yes | | ref | 0.95 (0.78-1.17) | 1.17 (0.97-1.43) | 1.09 (0.90-1.32) |  |
| No | | ref | 1.03 (0.86-1.24) | 1.12 (0.93-1.34) | 1.19 (0.99-1.42) |  |
| Cancer | |  |  |  |  | 0.938 |
| Yes | | ref | 0.80 (0.60-1.07) | 1.21 (0.92-1.59) | 1.02 (0.79-1.33) |  |
| No | | ref | 1.06 (0.91-1.25) | 1.14 (0.97-1.33) | **1.18 (1.01-1.38)** |  |
| CKD stage | |  |  |  |  | 0.535 |
| 1 | | ref | 1.29 (0.85-1.96) | **1.81 (1.22-2.69)** | 1.09 (0.72-1.66) |  |
| 2 | | ref | 0.81 (0.60-1.09) | 1.14 (0.87-1.51) | 1.18 (0.90-1.55) |  |
| 3 | | ref | 1.05 (0.87-1.27) | 1.09 (0.90-1.31) | 1.14 (0.96-1.36) |  |
| 4 | | ref | 0.92 (0.63-1.33) | 1.30 (0.86-1.97) | 1.13 (0.73-1.74) |  |
| ACR | |  |  |  |  | 0.643 |
| ≦30mg/g | | ref | 1.10 (0.88-1.37) | 1.12 (0.90-1.41) | 1.12 (0.91-1.38) |  |
| ﹥30mg/g | | ref | 0.92 (0.77-1.10) | **1.19 (1.00-1.41)** | 1.15 (0.97-1.37) |  |
| Hemoglobin |  | |  |  |  |  |
| Male: ﹤13.0 g/dL | ref | | 1.27 (0.91-1.79) | 1.32 (0.93-1.89) | 1.13 (0.79-1.62) | **0.031** |
| Male: ≧13.0 g/dL | ref | | 1.00 (0.80-1.25) | 1.09 (0.87-1.36) | **1.28 (1.04-1.58)** |  |
| Female: ﹤12.0 g/dL | ref | | 0.79 (0.50-1.23) | 1.16 (0.76-1.77) | 0.93 (0.61-1.41) | 0.480 |
| Female: ≧12.0 g/dL | ref | | 1.01 (0.79-1.28) | 1.19 (0.94-1.49) | 1.11 (0.89-1.38) |  |
| Serum phosphorus |  | |  |  |  | 0.111 |
| ≦3.05 mg/dL | ref | | 0.87 (0.56-1.36) | 1.21 (0.76-1.93) | 1.21 (0.76-1.92) |  |
| 3.06-4.45 mg/dL | ref | | 0.99 (0.85-1.16) | 1.12 (0.96-1.30) | 1.13 (0.97-1.30) |  |
| ﹥4.46 mg/dL | ref | | 1.05 (0.68-1.60) | 1.29 (0.85-1.95) | 1.02 (0.67-1.55) |  |

Adjusted covariates: age, sex, family income-poverty ratio level, race, education level, marital status, alcohol consumption, smoking, and leisure-time physical activity, log-transformed total energy intake, HEI-2015, baseline eGFR, log-transformed urinary ACR, body mass index, total-to-HDL cholesterol ratio, serum phosphorus, hemoglobin, hypertension, and diabetes, and history of cardiovascular disease and cancer. The variable used for stratification was not included in the given model. Interaction was tested using a continuous total carotene intake term and the exposure of interest, using a Wald test for dichotomous variables.

*Q* quintile, BMI body mass index, CVD cardiovascular disease, CKD chronic kidney disease, ACR albumin-Cr ratio.

**Supplementary Table 4** Subgroup analyses of the associations between lycopene intake and mortality.

| subgroups | Lycopene intake (mcg/kg per day) | | | | p value for interaction |
| --- | --- | --- | --- | --- | --- |
|  | Q1 (≦2.43) | Q2 (2.44-20.19) | Q3 (20.20-67.59) | Q4 (≧67.60) |  |
| Age |  |  |  |  | **0.024** |
| ﹤60 years | ref | 0.94 (0.60-1.46) | 0.80 (0.53-1.22) | 1.11 (0.75-1.66) |  |
| ≧60 years | ref | 0.90 (0.79-1.03) | 0.96 (0.84-1.10) | **0.81 (0.70-0.93)** |  |
| Sex |  |  |  |  | **0.031** |
| Male | ref | 0.92 (0.78-1.10) | 0.99 (0.83-1.18) | 0.91 (0.77-1.09) |  |
| Female | ref | 0.97 (0.81-1.17) | 0.95 (0.79-1.15) | 0.84 (0.69-1.02) |  |
| Race |  |  |  |  | **0.002** |
| Non-Hispanic white | ref | 0.94 (0.80-1.10) | 0.95 (0.81-1.11) | 0.81 (0.69-0.94) |  |
| Others | ref | 0.91 (0.74-1.12) | 0.93 (0.75-1.16) | 0.97 (0.77-1.23) |  |
| Education levels |  |  |  |  | **0.045** |
| ﹤High school | ref | 0.90 (0.77-1.04) | 1.04 (0.90-1.22) | 0.90 (0.77-1.06) |  |
| ≧High school | ref | 0.99 (0.80-1.23) | **0.78 (0.62-0.97)** | **0.77 (0.62-0.95)** |  |
| Marital status |  |  |  |  | 0.941 |
| Married | ref | 0.97 (0.80-1.17) | 0.99 (0.81-1.21) | 0.89 (0.73-1.08) |  |
| Unmarried | ref | 0.92 (0.78-1.08) | 0.96 (0.81-1.13) | 0.84 (0.71-1.00) |  |
| BMI |  |  |  |  | 0.872 |
| ﹤25 kg/m^2^ | ref | 1.03 (0.82-1.29) | 0.95 (0.75-1.20) | 0.90 (0.72-1.13) |  |
| ≧25 kg/m^2^ | ref | 0.88 (0.76-1.02) | 0.95 (0.82-1.11) | **0.84 (0.72-0.98)** |  |
| Alcohol drinking status |  |  |  |  | 0.191 |
| Abstainer | ref | 0.92 (0.77-1.00) | 1.02 (0.85-1.24) | 0.87 (0.71-1.05) |  |
| Drinker | ref | 0.95 (0.80-1.13) | 0.95 (0.80-1.12) | 0.87 (0.74-1.04) |  |
| Smoking |  |  |  |  | **<0.001** |
| Yes | ref | 0.96 (0.82-1.14) | 1.01 (0.86-1.19) | 0.91 (0.76-1.07) |  |
| No | ref | 0.87 (0.72-1.05) | 0.90 (0.74-1.10) | **0.79 (0.65-0.96)** |  |
| Hypertension |  |  |  |  | 0.888 |
| Yes | ref | 0.94 (0.82-1.07) | 0.95 (0.83-1.09) | 0.87 (0.75-1.00) |  |
| No | ref | 0.91 (0.66-1.24) | 1.02 (0.75-1.39) | 0.88 (0.64-1.21) |  |
| Diabetes |  |  |  |  | **0.001** |
| Yes | ref | 1.02 (0.82-1.28) | 1.10 (0.87-1.39) | 1.00 (0.79-1.27) |  |
| No | ref | 0.90 (0.77-1.04) | 0.91 (0.78-1.06) | **0.81 (0.70-0.95)** |  |
| CVD |  |  |  |  | 0.561 |
| Yes | ref | 0.95 (0.79-1.15) | 1.03 (0.86-1.25) | 0.85 (0.70-1.03) |  |
| No | ref | 0.89 (0.75-1.05) | 0.88 (0.74-1.05) | 0.88 (0.74-1.05) |  |
| Cancer |  |  |  |  | 0.734 |
| Yes | ref | 0.97 (0.75-1.25) | 1.09 (0.84-1.41) | **0.76 (0.58-0.99)** |  |
| No | ref | 0.94 (0.81-1.08) | 0.93 (0.80-1.08) | 0.91 (0.78-1.06) |  |
| CKD stage |  |  |  |  | 0.465 |
| 1 | ref | 0.84 (0.56-1.26) | 0.72 (0.46-1.11) | 0.81 (0.54-1.21) |  |
| 2 | ref | 0.88 (0.68-1.14) | 0.94 (0.72-1.22) | **0.74 (0.57-0.96)** |  |
| 3 | ref | 0.96 (0.81-1.13) | 0.98 (0.83-1.16) | 0.88 (0.74-1.04) |  |
| 4 | ref | 0.92 (0.63-1.33) | 1.30 (0.86-1.97) | 1.13 (0.73-1.74) |  |
| ACR |  |  |  |  | 0.054 |
| ≦30mg/g | ref | 1.01 (0.83-1.23) | 1.01 (0.82-1.23) | 0.91 (0.74-1.12) |  |
| ﹥30mg/g | ref | 0.89 (0.76-1.04) | 0.93 (0.79-1.09) | 0.85 (0.72-1.01) |  |
| Hemoglobin |  |  |  |  |  |
| Male: ﹤13.0 g/dL | ref | 1.01 (0.74-1.37) | 0.79 (0.56-1.11) | 0.85 (0.60-1.22) | 0.298 |
| Male: ≧13.0 g/dL | ref | 0.89 (0.72-1.10) | 1.08 (0.87-1.32) | 0.95 (0.78-1.16) |  |
| Female: ﹤12.0 g/dL | ref | 0.90 (0.61-1.33) | 1.24 (0.83-1.85) | 0.76 (0.48-1.22) | 0.057 |
| Female: ≧12.0 g/dL | ref | 0.97 (0.79-1.19) | 0.86 (0.69-1.06) | 0.84 (0.68-1.04) |  |
| Serum phosphorus |  |  |  |  | 0.047 |
| ≦3.05 mg/dL | ref | 0.98 (0.65-1.49) | 1.04 (0.68-1.59) | 0.75 (0.47-1.20) |  |
| 3.06-4.45 mg/dL | ref | 0.89 (0.78-1.02) | 0.91 (0.79-1.05) | **0.83 (0.72-0.95)** |  |
| ﹥4.46 mg/dL | ref | 1.18 (0.79-1.78) | **1.57 (1.06-2.33)** | 1.29 (0.87-1.90) |  |

Adjusted covariates: age, sex, family income-poverty ratio level, race, education level, marital status, alcohol consumption, smoking, and leisure-time physical activity, log-transformed total energy intake, HEI-2015, baseline eGFR, log-transformed urinary ACR, body mass index, total-to-HDL cholesterol ratio, serum phosphorus, hemoglobin, hypertension, and diabetes, and history of cardiovascular disease and cancer. The variable used for stratification was not included in the given model. Interaction was tested using a continuous total carotene intake term and the exposure of interest, using a Wald test for dichotomous variables.

*Q* quintile, BMI body mass index, CVD cardiovascular disease, CKD chronic kidney disease, ACR albumin-Cr ratio.

**Supplementary Table 5** Subgroup analyses of the associations between lutein + zeaxanthin intakes and mortality.

| subgroups | Lutein + zeaxanthin intake (mcg/kg per day) | | | | p value for interaction |
| --- | --- | --- | --- | --- | --- |
|  | Q1 (≦5.06) | Q2 (5.07-9.51) | Q3 (9.52-18.47) | Q4 (≧18.48) |  |
| Age |  |  |  |  | 0.180 |
| ﹤60 years | ref | 1.35 (0.90-2.02) | 1.08 (0.71-1.65) | 1.33 (0.85-2.08) |  |
| ≧60 years | ref | 1.03 (0.90-1.18) | 1.10 (0.96-1.26) | 1.03 (0.89-1.18) |  |
| Sex |  |  |  |  | 0.455 |
| Male | ref | 0.98 (0.82-1.17) | 1.11 (0.93-1.33) | 1.05 (0.86-1.27) |  |
| Female | ref | 0.94 (0.77-1.14) | 0.96 (0.78-1.17) | 0.88 (0.72-1.08) |  |
| Race |  |  |  |  | 0.698 |
| Non-Hispanic white | ref | 0.95 (0.81-1.12) | 1.04 (0.88-1.23) | 0.97 (0.81-1.16) |  |
| Others | ref | 0.97 (0.78-1.21) | 1.04 (0.83-1.32) | 1.03 (0.82-1.30) |  |
| Education levels |  |  |  |  | 0.529 |
| ﹤High school | ref | 0.96 (0.82-1.12) | 1.00 (0.85-1.18) | 0.99 (0.83-1.18) |  |
| ≧High school | ref | 0.96 (0.76-1.23) | 1.14 (0.89-1.45) | 0.93 (0.72-1.19) |  |
| Marital status |  |  |  |  | 0.535 |
| Married | ref | 0.95 (0.78-1.16) | 0.97 (0.79-1.18) | 1.12 (0.91-1.38) |  |
| Unmarried | ref | 0.96 (0.81-1.14) | 1.07 (0.91-1.27) | 0.86 (0.73-1.02) |  |
| BMI |  |  |  |  | 0.914 |
| ﹤25 kg/m^2^ | ref | 0.92 (0.71-1.20) | 1.10 (0.86-1.41) | 0.86 (0.67-1.12) |  |
| ≧25 kg/m^2^ | ref | 0.98 (0.84-1.14) | 1.00 (0.86-1.18) | 1.06 (0.89-1.25) |  |
| Alcohol drinking status |  |  |  |  | 0.149 |
| Abstainer | ref | 0.93 (0.77-1.13) | 0.97 (0.8-1.18) | 0.95 (0.77-1.16) |  |
| Drinker | ref | 0.97 (0.81-1.16) | 1.07 (0.9-1.28) | 0.97 (0.80-1.17) |  |
| Smoking |  |  |  |  | 0.327 |
| Yes | ref | 1.11 (0.94-1.32) | 1.11 (0.93-1.32) | 1.01 (0.84-1.22) |  |
| No | ref | **0.77 (0.63-0.94)** | 0.94 (0.77-1.15) | 0.86 (0.70-1.04) |  |
| Hypertension |  |  |  |  | 0.089 |
| Yes | ref | 0.96 (0.83-1.11) | 1.00 (0.86-1.16) | 0.99 (0.85-1.16) |  |
| No | ref | 1.09 (0.78-1.52) | 1.28 (0.93-1.77) | 0.90 (0.63-1.28) |  |
| Diabetes |  |  |  |  | 0.984 |
| Yes | ref | 1.10 (0.87-1.38) | 1.07 (0.85-1.37) | 1.05 (0.81-1.36) |  |
| No | ref | 0.89 (0.76-1.04) | 1.02 (0.87-1.19) | 0.92 (0.78-1.09) |  |
| CVD |  |  |  |  | 0.313 |
| Yes | ref | 1.05 (0.87-1.27) | 1.06 (0.87-1.28) | 0.92 (0.75-1.13) |  |
| No | ref | 0.88 (0.74-1.06) | 1.02 (0.85-1.22) | 1.03 (0.85-1.24) |  |
| Cancer |  |  |  |  | **0.018** |
| Yes | ref | 0.75 (0.57-1.00) | 0.87 (0.66-1.14) | 0.92 (0.70-1.21) |  |
| No | ref | 1.02 (0.88-1.19) | 1.07 (0.92-1.25) | 0.96 (0.81-1.14) |  |
| CKD stage |  |  |  |  | 0.379 |
| 1 | ref | 1.41 (0.93-2.12) | 1.25 (0.84-1.86) | 1.35 (0.88-2.07) |  |
| 2 | ref | 0.95 (0.71-1.27) | 0.99 (0.74-1.33) | 1.04 (0.77-1.40) |  |
| 3 | ref | 0.93 (0.78-1.10) | 1.05 (0.88-1.25) | 0.95 (0.78-1.14) |  |
| 4 | ref | 1.08 (0.74-1.57) | 0.86 (0.56-1.32) | 0.73 (0.47-1.15) |  |
| ACR |  |  |  |  | 0.903 |
| ≦30mg/g | ref | 1.05 (0.84-1.30) | 1.03 (0.82-1.28) | 0.95 (0.75-1.20) |  |
| ﹥30mg/g | ref | 0.92 (0.78-1.09) | 1.04 (0.87-1.23) | 1.02 (0.85-1.22) |  |
| Hemoglobin |  |  |  |  |  |
| Male: ﹤13.0 g/dL | ref | 0.95 (0.67-1.33) | 1.03 (0.73-1.45) | 0.75 (0.50-1.13) | **0.020** |
| Male: ≧13.0 g/dL | ref | 1.01 (0.82-1.25) | 1.15 (0.93-1.42) | 1.19 (0.95-1.48) |  |
| Female: ﹤12.0 g/dL | ref | 0.73 (0.47-1.13) | 1.04 (0.65-1.66) | 0.99 (0.63-1.54) | **<0.001** |
| Female: ≧12.0 g/dL | ref | 0.95 (0.76-1.18) | 0.94 (0.76-1.17) | 0.83 (0.66-1.03) |  |
| Serum phosphorus |  |  |  |  | 0.562 |
| ≦3.05 mg/dL | ref | 0.74 (0.47-1.15) | **0.66 (0.44-0.98)** | 1.04 (0.70-1.54) |  |
| 3.06-4.45 mg/dL | ref | 0.92 (0.79-1.06) | 1.05 (0.91-1.21) | 0.88 (0.76-1.02) |  |
| ﹥4.46 mg/dL | ref | 1.33 (0.89-1.98) | 1.25 (0.81-1.94) | 1.44 (0.91-2.28) |  |

Adjusted covariates: age, sex, family income-poverty ratio level, race, education level, marital status, alcohol consumption, smoking, and leisure-time physical activity, log-transformed total energy intake, HEI-2015, baseline eGFR, log-transformed urinary ACR, body mass index, total-to-HDL cholesterol ratio, serum phosphorus, hemoglobin, hypertension, and diabetes, and history of cardiovascular disease and cancer. The variable used for stratification was not included in the given model. Interaction was tested using a continuous total carotene intake term and the exposure of interest, using a Wald test for dichotomous variables.

*Q* quintile, BMI body mass index, CVD cardiovascular disease, CKD chronic kidney disease, ACR albumin-Cr ratio.

**Supplementary Table 6** Pearson correlation analyses between carotene intakes and its correspondence serum level

|  | Pearson correlation coefficient | P |
| --- | --- | --- |
| Alpha-carotene intake V.S. serum alpha-carotene | 0.342 | <0.001 |
| Beta-carotene intake V.S. serum beta-carotene | 0.288 | <0.001 |
| Beta-cryptoxanthin intake V.S. serum beta-cryptoxanthin | 0.391 | <0.001 |
| Lycopene intake V.S. serum lycopene | 0.175 | <0.001 |
| Lutein + zeaxanthin intake V.S. serum lutein + zeaxanthin | 0.302 | <0.001 |

**Supplementary Table 7** Subgroup analyses of the associations between serum alpha-carotene level and mortality.

| subgroups | Alpha-carotene (ug/dL) | | | | p value for interaction |
| --- | --- | --- | --- | --- | --- |
|  | Q1 (≦1.50) | Q2 (1.51-2.80) | Q3 (2.81-5.20) | Q4 (≧5.21) |  |
| Age |  |  |  |  | **<0.001** |
| ﹤60 years | ref | 0.80 (0.49-1.28) | 0.72 (0.39-1.32) | 0.70 (0.36-1.36) |  |
| ≧60 years | ref | 0.93 (0.78-1.11) | 0.93 (0.78-1.11) | 0.92 (0.76-1.11) |  |
| Sex |  |  |  |  | 0.535 |
| Male | ref | 0.89 (0.71-1.11) | 0.80 (0.64-1.00) | 0.79 (0.61-1.02) |  |
| Female | ref | 0.89 (0.69-1.14) | 0.79 (0.61-1.00) | **0.76 (0.60-0.97)** |  |
| Race |  |  |  |  | **0.002** |
| Non-Hispanic white | ref | 0.89 (0.73-1.10) | 0.84 (0.69-1.03) | 0.84 (0.69-1.04) |  |
| Others | ref | 0.82 (0.61-1.09) | **0.71 (0.53-0.95)** | **0.64 (0.46-0.89)** |  |
| Education levels |  |  |  |  | **0.003** |
| ﹤High school | ref | 0.86 (0.70-1.05) | 0.83 (0.68-1.01) | **0.79 (0.64-0.98)** |  |
| ≧High school | ref | 0.80 (0.58-1.09) | **0.66 (0.49-0.91)** | **0.71 (0.51-0.98)** |  |
| Marital status |  |  |  |  | 0.756 |
| Married | ref | 0.97 (0.76-1.24) | 0.78 (0.61-1.01) | 0.87 (0.66-1.16) |  |
| Unmarried | ref | **0.79 (0.64-0.99)** | **0.80 (0.64-0.99)** | **0.71 (0.57-0.89)** |  |
| BMI |  |  |  |  | **0.042** |
| ﹤25 kg/m^2^ | ref | **0.66 (0.48-0.91)** | **0.66 (0.48-0.92)** | **0.52 (0.37-0.72)** |  |
| ≧25 kg/m^2^ | ref | 0.97 (0.79-1.18) | 0.84 (0.69-1.02) | 0.87 (0.71-1.07) |  |
| Alcohol drinking status |  |  |  |  | **<0.001** |
| Abstainer | ref | 0.90 (0.71-1.16) | 0.81 (0.63-1.04) | 0.80 (0.62-1.04) |  |
| Drinker | ref | 0.88 (0.70-1.10) | **0.78 (0.63-0.98)** | 0.79 (0.62-1.00) |  |
| Smoking |  |  |  |  | **0.002** |
| Yes | ref | 0.91 (0.74-1.12) | 0.81 (0.66-1.01) | 0.85 (0.67-1.09) |  |
| No | ref | 0.82 (0.62-1.08) | 0.71 (0.54-0.92) | **0.65 (0.50-0.85)** |  |
| Hypertension |  |  |  |  | 0.066 |
| Yes | ref | 0.90 (0.76-1.08) | **0.81 (0.68-0.98)** | **0.79 (0.65-0.96)** |  |
| No | ref | 0.69 (0.44-1.09) | 0.67 (0.45-1.01) | 0.70 (0.46-1.08) |  |
| Diabetes |  |  |  |  | 0.097 |
| Yes | ref | 0.95 (0.72-1.26) | **0.69 (0.50-0.95)** | **0.68 (0.48-0.96)** |  |
| No | ref | 0.85 (0.69-1.05) | 0.85 (0.70-1.04) | 0.81 (0.66-1.00) |  |
| CVD |  |  |  |  | 0.792 |
| Yes | ref | 0.90 (0.71-1.14) | 0.81 (0.63-1.03) | 0.89 (0.68-1.16) |  |
| No | ref | 0.82 (0.65-1.04) | **0.76 (0.60-0.96)** | **0.70 (0.55-0.88)** |  |
| Cancer |  |  |  |  | 0.704 |
| Yes | ref | 0.81 (0.56-1.16) | 0.78 (0.55-1.12) | 0.77 (0.52-1.15) |  |
| No | ref | 0.89 (0.74-1.08) | **0.80 (0.66-0.96)** | **0.78 (0.64-0.94)** |  |
| CKD stage |  |  |  |  | 0.882 |
| 1 | ref | 0.81 (0.45-1.44) | 1.00 (0.54-1.85) | 0.60 (0.26-1.39) |  |
| 2 | ref | **0.68 (0.48-0.95)** | **0.62 (0.44-0.89)** | **0.58 (0.40-0.84)** |  |
| 3 | ref | 0.98 (0.79-1.23) | **0.79 (0.64-0.99)** | 0.84 (0.67-1.06) |  |
| 4 | ref | 0.66 (0.34-1.30) | 0.91 (0.52-1.59) | 0.87 (0.46-1.65) |  |
| ACR |  |  |  |  | 0.651 |
| ≦30mg/g | ref | 0.92 (0.70-1.20) | 0.82 (0.63-1.07) | 0.77 (0.59-1.01) |  |
| ﹥30mg/g | ref | 0.82 (0.66-1.01) | **0.76 (0.62-0.95)** | **0.77 (0.61-0.97)** |  |
| Hemoglobin |  |  |  |  |  |
| Male: ﹤13.0 g/dL | ref | 0.60 (0.35-1.03) | 0.88 (0.55-1.41) | 0.61 (0.33-1.14) | 0.881 |
| Male: ≧13.0 g/dL | ref | 0.99 (0.78-1.27) | 0.78 (0.60-1.01) | 0.86 (0.65-1.15) |  |
| Female: ﹤12.0 g/dL | ref | **0.51 (0.27-0.96)** | **0.32 (0.16-0.62)** | **0.47 (0.25-0.90)** | 0.486 |
| Female: ≧12.0 g/dL | ref | 0.93 (0.71-1.23) | 0.91 (0.69-1.21) | 0.83 (0.62-1.10) |  |
| Serum phosphorus |  |  |  |  | **0.001** |
| ≦3.05 mg/dL | ref | 1.43 (0.86-2.40) | 0.75 (0.43-1.31) | 0.98 (0.55-1.74) |  |
| 3.06-4.45 mg/dL | ref | 0.84 (0.70-1.01) | 0.80 (0.67-0.97) | 0.75 (0.62-0.91) |  |
| ﹥4.46 mg/dL | ref | 0.53 (0.32-0.88) | 0.32 (0.19-0.56) | 0.52 (0.30-0.88) |  |

Adjusted covariates: age, sex, family income-poverty ratio level, race, education level, marital status, alcohol consumption, smoking, and leisure-time physical activity, log-transformed total energy intake, HEI-2015, baseline eGFR, log-transformed urinary ACR, body mass index, total-to-HDL cholesterol ratio, serum phosphorus, hemoglobin, hypertension, and diabetes, and history of cardiovascular disease and cancer. The variable used for stratification was not included in the given model. Interaction was tested using a continuous total carotene intake term and the exposure of interest, using a Wald test for dichotomous variables.

*Q* quintile, BMI body mass index, CVD cardiovascular disease, CKD chronic kidney disease, ACR albumin-Cr ratio.

**Supplementary Table 8** Subgroup analyses of the associations between serum beta-carotene level and mortality.

| subgroups | Beta-carotene (ug/dL) | | | | p value for interaction |
| --- | --- | --- | --- | --- | --- |
|  | Q1 (≦8.49) | Q2 (8.50-15.29) | Q3 (15.30-27.02) | Q4 (≧27.03) |  |
| Age |  |  |  |  | **<0.001** |
| ﹤60 years | ref | 1.07 (0.69-1.68) | 1.37 (0.77-2.42) | 0.73 (0.28-1.91) |  |
| ≧60 years | ref | 0.86 (0.71-1.03) | 0.91 (0.75-1.09) | 1.00 (0.83-1.21) |  |
| Sex |  |  |  |  | 0.116 |
| Male | ref | 0.83 (0.67-1.04) | 0.81 (0.64-1.03) | 0.94 (0.74-1.20) |  |
| Female | ref | **0.61 (0.46-0.80)** | **0.72 (0.54-0.94)** | **0.65 (0.49-0.86)** |  |
| Race |  |  |  |  | **0.001** |
| Non-Hispanic white | ref | **0.75 (0.60-0.93)** | **0.73 (0.59-0.91)** | 0.82 (0.66-1.02) |  |
| Others | ref | **0.74 (0.55-0.99)** | 0.98 (0.73-1.33) | 0.77 (0.55-1.08) |  |
| Education levels |  |  |  |  | 0.158 |
| ﹤High school | ref | **0.74 (0.60-0.90)** | **0.78 (0.63-0.97)** | 0.80 (0.65-1.00) |  |
| ≧High school | ref | 0.82 (0.58-1.15) | 0.90 (0.64-1.26) | 0.88 (0.63-1.23) |  |
| Marital status |  |  |  |  | 0.086 |
| Married | ref | **0.73 (0.56-0.95)** | **0.70 (0.53-0.91)** | 0.96 (0.73-1.26) |  |
| Unmarried | ref | **0.78 (0.62-0.99)** | 0.90 (0.71-1.14) | **0.76 (0.60-0.97)** |  |
| BMI |  |  |  |  | 0.593 |
| ﹤25 kg/m^2^ | ref | 0.71 (0.50-1.00) | 0.74 (0.53-1.04) | **0.63 (0.45-0.88)** |  |
| ≧25 kg/m^2^ | ref | 1.01 (0.83-1.21) | 0.91 (0.75-1.10) | 0.87 (0.71-1.07) |  |
| Alcohol drinking status |  |  |  |  | **0.012** |
| Abstainer | ref | **0.70 (0.54-0.92)** | 0.78 (0.60-1.02) | **0.70 (0.53-0.92)** |  |
| Drinker | ref | **0.77 (0.61-0.96)** | **0.78 (0.61-0.99)** | 0.89 (0.70-1.13) |  |
| Smoking |  |  |  |  | **0.023** |
| Yes | ref | 0.88 (0.71-1.09) | 0.84 (0.67-1.06) | 0.91 (0.72-1.15) |  |
| No | ref | **0.54 (0.40-0.73)** | **0.67 (0.50-0.90)** | **0.62 (0.46-0.84)** |  |
| Hypertension |  |  |  |  | **0.009** |
| Yes | ref | **0.81 (0.67-0.97)** | 0.86 (0.71-1.04) | 0.89 (0.73-1.08) |  |
| No | ref | **0.50 (0.32-0.77)** | **0.47 (0.30-0.72)** | **0.49 (0.31-0.77)** |  |
| Diabetes |  |  |  |  | 0.424 |
| Yes | ref | 0.79 (0.58-1.08) | 0.85 (0.61-1.18) | 0.80 (0.56-1.14) |  |
| No | ref | **0.78 (0.63-0.96)** | **0.78 (0.63-0.97)** | 0.81 (0.66-1.01) |  |
| CVD |  |  |  |  | 0.562 |
| Yes | ref | 0.82 (0.64-1.06) | 0.86 (0.67-1.12) | 0.89 (0.68-1.15) |  |
| No | ref | **0.71 (0.55-0.90)** | **0.77 (0.60-0.98)** | **0.76 (0.59-0.98)** |  |
| Cancer |  |  |  |  | **0.011** |
| Yes | ref | **0.55 (0.38-0.79)** | **0.57 (0.40-0.83)** | **0.52 (0.36-0.76)** |  |
| No | ref | 0.84 (0.69-1.02) | 0.88 (0.72-1.08) | 0.94 (0.77-1.16) |  |
| CKD stage |  |  |  |  | 0.127 |
| 1 | ref | 0.85 (0.48-1.48) | 0.65 (0.34-1.23) | 0.55 (0.21-1.39) |  |
| 2 | ref | 0.80 (0.56-1.14) | 0.81 (0.55-1.19) | 0.78 (0.53-1.15) |  |
| 3 | ref | 0.81 (0.64-1.03) | 0.86 (0.68-1.10) | 0.93 (0.73-1.17) |  |
| 4 | ref | 0.61 (0.33-1.15) | 0.78 (0.42-1.45) | **0.53 (0.28-0.98)** |  |
| ACR |  |  |  |  | 0.837 |
| ≦30mg/g | ref | **0.67 (0.50-0.90)** | **0.67 (0.51-0.88)** | **0.68 (0.52-0.89)** |  |
| ﹥30mg/g | ref | 0.84 (0.68-1.05) | 0.88 (0.69-1.11) | 0.91 (0.72-1.17) |  |
| Hemoglobin |  |  |  |  |  |
| Male: ﹤13.0 g/dL | ref | 0.70 (0.42-1.16) | 0.74 (0.43-1.27) | 0.98 (0.58-1.65) | 0.352 |
| Male: ≧13.0 g/dL | ref | 0.88 (0.68-1.13) | 0.82 (0.62-1.07) | 0.94 (0.71-1.25) |  |
| Female: ﹤12.0 g/dL | ref | 0.69 (0.30-1.63) | 0.59 (0.25-1.35) | 0.54 (0.23-1.25) | 0.871 |
| Female: ≧12.0 g/dL | ref | **0.59 (0.43-0.80)** | **0.70 (0.51-0.94)** | **0.66 (0.49-0.89)** |  |
| Serum phosphorus |  |  |  |  | 0.086 |
| ≦3.05 mg/dL | ref | 0.63 (0.37-1.07) | **0.57 (0.34-0.96)** | 0.75 (0.42-1.34) |  |
| 3.06-4.45 mg/dL | ref | **0.77 (0.63-0.93)** | **0.78 (0.63-0.95)** | **0.80 (0.65-0.98)** |  |
| ﹥4.46 mg/dL | ref | 0.62 (0.36-1.07) | 0.80 (0.45-1.43) | 0.92 (0.49-1.73) |  |

Adjusted covariates: age, sex, family income-poverty ratio level, race, education level, marital status, alcohol consumption, smoking, and leisure-time physical activity, log-transformed total energy intake, HEI-2015, baseline eGFR, log-transformed urinary ACR, body mass index, total-to-HDL cholesterol ratio, serum phosphorus, hemoglobin, hypertension, and diabetes, and history of cardiovascular disease and cancer. The variable used for stratification was not included in the given model. Interaction was tested using a continuous total carotene intake term and the exposure of interest, using a Wald test for dichotomous variables.

*Q* quintile, BMI body mass index, CVD cardiovascular disease, CKD chronic kidney disease, ACR albumin-Cr ratio.

**Supplementary Table 9** Subgroup analyses of the associations between serum beta- cryptoxanthin level and mortality.

| subgroups | Beta-cryptoxanthin (ug/dL) | | | | p value for interaction |
| --- | --- | --- | --- | --- | --- |
|  | Q1 (≦5.00) | Q2 (5.01-8.62) | Q3 (8.63-13.44) | Q4 (≧13.45) |  |
| Age |  |  |  |  | **0.007** |
| ﹤60 years | ref | 0.91 (0.56-1.45) | 0.57 (0.31-1.05) | 1.00 (0.54-1.86) |  |
| ≧60 years | ref | 0.91 (0.77-1.07) | 0.97 (0.82-1.15) | 0.91 (0.76-1.09) |  |
| Sex |  |  |  |  | 0.993 |
| Male | ref | 1.07 (0.86-1.32) | 0.89 (0.71-1.11) | 0.90 (0.71-1.14) |  |
| Female | ref | 0.82 (0.65-1.04) | 0.81 (0.65-1.02) | **0.72 (0.57-0.91)** |  |
| Race |  |  |  |  | **0.006** |
| Non-Hispanic white | ref | 0.87 (0.73-1.05) | 0.88 (0.73-1.06) | 0.85 (0.70-1.04) |  |
| Others | ref | 1.07 (0.80-1.44) | 0.83 (0.61-1.12) | 0.76 (0.56-1.04) |  |
| Education levels |  |  |  |  | 0.696 |
| ﹤High school | ref | 0.97 (0.80-1.17) | 0.90 (0.74-1.09) | 0.86 (0.70-1.05) |  |
| ≧High school | ref | 0.92 (0.69-1.22) | 0.80 (0.60-1.06) | 0.77 (0.57-1.05) |  |
| Marital status |  |  |  |  | **0.009** |
| Married | ref | 1.14 (0.90-1.45) | 1.02 (0.79-1.31) | 1.09 (0.83-1.42) |  |
| Unmarried | ref | 0.83 (0.67-1.03) | 0.84 (0.68-1.03) | **0.74 (0.59-0.92)** |  |
| BMI |  |  |  |  | 0.478 |
| ﹤25 kg/m^2^ | ref | 0.75 (0.56-1.01) | **0.68 (0.51-0.92)** | **0.61 (0.46-0.83)** |  |
| ≧25 kg/m^2^ | ref | 1.01 (0.83-1.21) | 0.91 (0.75-1.10) | 0.87 (0.71-1.07) |  |
| Alcohol drinking status |  |  |  |  | 0.124 |
| Abstainer | ref | 0.91 (0.72-1.14) | 0.82 (0.65-1.04) | **0.74 (0.57-0.95)** |  |
| Drinker | ref | 0.98 (0.79-1.21) | 0.92 (0.74-1.14) | 0.96 (0.76-1.21) |  |
| Smoking |  |  |  |  | **0.011** |
| Yes | ref | 0.90 (0.73-1.10) | 0.94 (0.76-1.17) | 0.93 (0.74-1.17) |  |
| No | ref | 1.11 (0.86-1.42) | 0.84 (0.66-1.08) | **0.73 (0.57-0.95)** |  |
| Hypertension |  |  |  |  | **0.040** |
| Yes | ref | 0.99 (0.84-1.18) | 0.93 (0.78-1.11) | 0.85 (0.71-1.03) |  |
| No | ref | 0.68 (0.46-1.01) | **0.58 (0.39-0.84)** | 0.70 (0.47-1.02) |  |
| Diabetes |  |  |  |  | 0.900 |
| Yes | ref | 1.14 (0.85-1.52) | 0.96 (0.71-1.30) | 0.83 (0.60-1.16) |  |
| No | ref | 0.89 (0.74-1.08) | 0.83 (0.68-1.00) | 0.83 (0.68-1.00) |  |
| CVD |  |  |  |  | 0.857 |
| Yes | ref | 1.06 (0.84-1.33) | 0.9 (0.71-1.14) | 0.94 (0.72-1.23) |  |
| No | ref | 0.85 (0.69-1.06) | 0.8 (0.65-1.00) | **0.76 (0.61-0.95)** |  |
| Cancer |  |  |  |  | 0.674 |
| Yes | ref | 0.88 (0.62-1.23) | **0.69 (0.49-0.95)** | 0.82 (0.58-1.17) |  |
| No | ref | 0.98 (0.82-1.17) | 0.93 (0.78-1.12) | 0.84 (0.69-1.02) |  |
| CKD stage |  |  |  |  | **<0.001** |
| 1 | ref | 0.61 (0.36-1.03) | 0.50 (0.24-1.03) | 0.60 (0.28-1.25) |  |
| 2 | ref | 0.75 (0.53-1.07) | 0.89 (0.63-1.26) | 0.79 (0.55-1.14) |  |
| 3 | ref | 1.02 (0.83-1.25) | 0.88 (0.72-1.09) | 0.82 (0.66-1.02) |  |
| 4 | ref | 1.52 (0.91-2.54) | 1.28 (0.75-2.18) | 1.24 (0.66-2.31) |  |
| ACR |  |  |  |  | **0.007** |
| ≦30mg/g | ref | 1.10 (0.86-1.40) | 0.84 (0.66-1.07) | 0.84 (0.65-1.09) |  |
| ﹥30mg/g | ref | 0.84 (0.69-1.03) | 0.84 (0.68-1.04) | **0.78 (0.63-0.98)** |  |
| Hemoglobin |  |  |  |  |  |
| Male: ﹤13.0 g/dL | ref | 1.03 (0.66-1.59) | 0.98 (0.59-1.62) | 0.47 (0.29-0.78) | 0.279 |
| Male: ≧13.0 g/dL | ref | 1.02 (0.80-1.31) | 0.89 (0.69-1.15) | 1.04 (0.79-1.36) |  |
| Female: ﹤12.0 g/dL | ref | 1.25 (0.64-2.48) | 0.72 (0.40-1.31) | 0.53 (0.25-1.09) | **0.002** |
| Female: ≧12.0 g/dL | ref | **0.76 (0.59-0.99)** | 0.79 (0.61-1.02) | **0.70 (0.54-0.90)** |  |
| Serum phosphorus |  |  |  |  | **0.030** |
| ≦3.05 mg/dL | ref | 1.08 (0.61-1.92) | 0.84 (0.48-1.47) | 0.96 (0.52-1.79) |  |
| 3.06-4.45 mg/dL | ref | 0.96 (0.80-1.14) | 0.89 (0.74-1.06) | 0.83 (0.69-1.00) |  |
| ﹥4.46 mg/dL | ref | 0.94 (0.56-1.58) | 0.66 (0.39-1.10) | 1.03 (0.60-1.76) |  |

Adjusted covariates: age, sex, family income-poverty ratio level, race, education level, marital status, alcohol consumption, smoking, and leisure-time physical activity, log-transformed total energy intake, HEI-2015, baseline eGFR, log-transformed urinary ACR, body mass index, total-to-HDL cholesterol ratio, serum phosphorus, hemoglobin, hypertension, and diabetes, and history of cardiovascular disease and cancer. The variable used for stratification was not included in the given model. Interaction was tested using a continuous total carotene intake term and the exposure of interest, using a Wald test for dichotomous variables.

*Q* quintile, BMI body mass index, CVD cardiovascular disease, CKD chronic kidney disease, ACR albumin-Cr ratio.

**Supplementary Table 10** Subgroup analyses of the associations between serum lycopene level and mortality.

| subgroups | Lycopene (ug/dL) | | | | p value for interaction |
| --- | --- | --- | --- | --- | --- |
|  | Q1 (≦14.90) | Q2 (14.91-25.50) | Q3 (25.51-38.64) | Q4 (≧38.65) |  |
| Age |  |  |  |  | **0.002** |
| ﹤60 years | ref | 1.31 (0.70-2.42) | 1.07 (0.59-1.94) | 1.00 (0.56-1.78) |  |
| ≧60 years | ref | **0.79 (0.68-0.92)** | **0.74 (0.63-0.87)** | **0.70 (0.59-0.84)** |  |
| Sex |  |  |  |  | 0.567 |
| Male | ref | **0.73 (0.60-0.91)** | **0.72 (0.58-0.89)** | **0.73 (0.58-0.93)** |  |
| Female | ref | 0.87 (0.70-1.08) | 0.90 (0.71-1.14) | 0.82 (0.64-1.04) |  |
| Race |  |  |  |  | 0.766 |
| Non-Hispanic white | ref | **0.76 (0.64-0.91)** | **0.73 (0.60-0.89)** | **0.81 (0.66-0.98)** |  |
| Others | ref | 0.86 (0.65-1.14) | 0.95 (0.72-1.27) | **0.68 (0.49-0.95)** |  |
| Education levels |  |  |  |  | **0.001** |
| ﹤High school | ref | **0.79 (0.66-0.94)** | 0.84 (0.69-1.02) | **0.75 (0.61-0.93)** |  |
| ≧High school | ref | 0.85 (0.65-1.11) | **0.68 (0.51-0.90)** | 0.79 (0.59-1.05) |  |
| Marital status |  |  |  |  | **0.002** |
| Married | ref | 0.89 (0.70-1.12) | 0.79 (0.62-1.01) | 0.93 (0.72-1.20) |  |
| Unmarried | ref | **0.76 (0.62-0.92)** | 0.84 (0.68-1.04) | **0.70 (0.56-0.89)** |  |
| BMI |  |  |  |  | 0.386 |
| ﹤25 kg/m^2^ | ref | 0.99 (0.76-1.30) | 0.85 (0.64-1.14) | 0.81 (0.59-1.12) |  |
| ≧25 kg/m^2^ | ref | 0.86 (0.71-1.04) | **0.68 (0.56-0.82)** | **0.81 (0.66-0.99)** |  |
| Alcohol drinking status |  |  |  |  | 0.219 |
| Abstainer | ref | 0.82 (0.66-1.03) | 0.85 (0.67-1.07) | **0.72 (0.56-0.92)** |  |
| Drinker | ref | 0.85 (0.69-1.04) | **0.76 (0.61-0.95)** | 0.87 (0.69-1.10) |  |
| Smoking |  |  |  |  | 0.181 |
| Yes | ref | 0.83 (0.68-1.02) | 0.87 (0.71-1.07) | 0.83 (0.66-1.04) |  |
| No | ref | 0.83 (0.67-1.04) | **0.71 (0.55-0.92)** | 0.79 (0.61-1.01) |  |
| Hypertension |  |  |  |  | **0.008** |
| Yes | ref | **0.79 (0.67-0.94)** | 0.85 (0.72-1.02) | **0.82 (0.68-0.98)** |  |
| No | ref | 0.83 (0.58-1.19) | **0.59 (0.39-0.89)** | 0.67 (0.43-1.02) |  |
| Diabetes |  |  |  |  | 0.569 |
| Yes | ref | **0.70 (0.52-0.94)** | 0.79 (0.58-1.08) | 0.73 (0.54-1.00) |  |
| No | ref | **0.82 (0.69-0.97)** | **0.77 (0.64-0.93)** | **0.77 (0.63-0.94)** |  |
| CVD |  |  |  |  | 0.400 |
| Yes | ref | 0.80 (0.64-1.00) | 0.87 (0.68-1.11) | 0.84 (0.65-1.09) |  |
| No | ref | **0.79 (0.64-0.97)** | **0.76 (0.61-0.94)** | **0.72 (0.58-0.91)** |  |
| Cancer |  |  |  |  | 0.076 |
| Yes | ref | 0.81 (0.59-1.12) | 0.92 (0.65-1.29) | 0.79 (0.55-1.14) |  |
| No | ref | **0.77 (0.65-0.92)** | **0.75 (0.63-0.90)** | **0.76 (0.62-0.92)** |  |
| CKD stage |  |  |  |  | 0.943 |
| 1 | ref | 1.01 (0.57-1.81) | **0.51 (0.27-0.95)** | 0.88 (0.47-1.65) |  |
| 2 | ref | **0.68 (0.49-0.94)** | **0.68 (0.49-0.96)** | **0.70 (0.49-0.99)** |  |
| 3 | ref | **0.80 (0.66-0.96)** | 0.82 (0.67-1.02) | **0.78 (0.63-0.97)** |  |
| 4 | ref | 0.83 (0.48-1.43) | 1.12 (0.66-1.91) | 1.09 (0.58-2.04) |  |
| ACR |  |  |  |  | 0.614 |
| ≦30mg/g | ref | 0.80 (0.64-1.00) | **0.76 (0.60-0.97)** | **0.74 (0.57-0.97)** |  |
| ﹥30mg/g | ref | **0.79 (0.65-0.97)** | **0.77 (0.62-0.95)** | 0.84 (0.68-1.05) |  |
| Hemoglobin |  |  |  |  |  |
| Male: ﹤13.0 g/dL | ref | 0.67 (0.43-1.05) | **0.55 (0.34-0.88)** | **0.51 (0.30-0.89)** | 0.138 |
| Male: ≧13.0 g/dL | ref | **0.74 (0.58-0.94)** | **0.76 (0.59-0.98)** | 0.79 (0.61-1.03) |  |
| Female: ﹤12.0 g/dL | ref | 0.66 (0.37-1.17) | 0.57 (0.31-1.03) | **0.32 (0.15-0.67)** | **0.019** |
| Female: ≧12.0 g/dL | ref | 0.89 (0.71-1.13) | 0.92 (0.70-1.19) | 0.91 (0.70-1.19) |  |
| Serum phosphorus |  |  |  |  | 0.424 |
| ≦3.05 mg/dL | ref | 0.94 (0.56-1.57) | 0.57 (0.31-1.04) | 0.85 (0.47-1.53) |  |
| 3.06-4.45 mg/dL | ref | **0.79 (0.67-0.93)** | **0.77 (0.64-0.92)** | **0.74 (0.62-0.90)** |  |
| ﹥4.46 mg/dL | ref | 0.75 (0.44-1.30) | 1.17 (0.68-2.01) | 1.07 (0.61-1.88) |  |

Adjusted covariates: age, sex, family income-poverty ratio level, race, education level, marital status, alcohol consumption, smoking, and leisure-time physical activity, log-transformed total energy intake, HEI-2015, baseline eGFR, log-transformed urinary ACR, body mass index, total-to-HDL cholesterol ratio, serum phosphorus, hemoglobin, hypertension, and diabetes, and history of cardiovascular disease and cancer. The variable used for stratification was not included in the given model. Interaction was tested using a continuous total carotene intake term and the exposure of interest, using a Wald test for dichotomous variables.

*Q* quintile, BMI body mass index, CVD cardiovascular disease, CKD chronic kidney disease, ACR albumin-Cr ratio.

**Supplementary Table 11** Subgroup analyses of the associations between serum lutein + zeaxanthin level and mortality.

| subgroups | Lutein + zeaxanthin (ug/dL) | | | | p value for interaction |
| --- | --- | --- | --- | --- | --- |
|  | Q1 (≦10.40) | Q2 (10.41-14.72) | Q3 (14.73-20.80) | Q4 (≧20.81) |  |
| Age |  |  |  |  | **0.002** |
| ﹤60 years | ref | 0.99 (0.61-1.61) | 0.64 (0.37-1.12) | 0.78 (0.42-1.45) |  |
| ≧60 years | ref | 0.91 (0.77-1.08) | **0.74 (0.62-0.88)** | 0.94 (0.80-1.11) |  |
| Sex |  |  |  |  | 0.639 |
| Male | ref | 0.86 (0.70-1.07) | **0.72 (0.57-0.90)** | 0.83 (0.66-1.04) |  |
| Female | ref | 0.85 (0.67-1.07) | **0.65 (0.51-0.82)** | 0.82 (0.65-1.03) |  |
| Race |  |  |  |  | 0.784 |
| Non-Hispanic white | ref | **0.81 (0.67-0.98)** | **0.68 (0.56-0.83)** | 0.91 (0.75-1.10) |  |
| Others | ref | 0.90 (0.67-1.22) | **0.68 (0.49-0.93)** | **0.70 (0.52-0.95)** |  |
| Education levels |  |  |  |  | **0.001** |
| ﹤High school | ref | 0.82 (0.68-1.00) | **0.71 (0.58-0.86)** | **0.79 (0.65-0.96)** |  |
| ≧High school | ref | 0.75 (0.57-1.00) | **0.57 (0.42-0.76)** | 0.76 (0.57-1.02) |  |
| Marital status |  |  |  |  | **<0.001** |
| Married | ref | **0.78 (0.62-0.99)** | **0.60 (0.47-0.78)** | 0.82 (0.64-1.05) |  |
| Unmarried | ref | 0.89 (0.72-1.10) | **0.77 (0.62-0.96)** | 0.84 (0.67-1.03) |  |
| BMI |  |  |  |  | **<0.001** |
| ﹤25 kg/m2 | ref | 0.76 (0.57-1.02) | **0.70 (0.51-0.98)** | 0.79 (0.58-1.05) |  |
| ≧25 kg/m2 | ref | 0.86 (0.71-1.04) | **0.68 (0.56-0.82)** | **0.81 (0.66-0.99)** |  |
| Alcohol drinking status |  |  |  |  | **0.001** |
| Abstainer | ref | **0.78 (0.62-0.98)** | **0.64 (0.50-0.82)** | **0.76 (0.60-0.97)** |  |
| Drinker | ref | 0.92 (0.74-1.15) | **0.71 (0.57-0.88)** | 0.88 (0.71-1.10) |  |
| Smoking |  |  |  |  | **<0.001** |
| Yes | ref | 0.93 (0.76-1.15) | **0.73 (0.59-0.91)** | 0.83 (0.67-1.04) |  |
| No | ref | **0.73 (0.57-0.94)** | **0.60 (0.46-0.77)** | **0.75 (0.59-0.96)** |  |
| Hypertension |  |  |  |  | **0.028** |
| Yes | ref | 0.85 (0.72-1.01) | **0.71 (0.60-0.86)** | 0.84 (0.70-1.00) |  |
| No | ref | 0.77 (0.53-1.12) | **0.58 (0.39-0.86)** | **0.66 (0.44-0.98)** |  |
| Diabetes |  |  |  |  | 0.379 |
| Yes | ref | 0.94 (0.71-1.26) | **0.64 (0.47-0.88)** | 0.80 (0.58-1.10) |  |
| No | ref | **0.80 (0.66-0.96)** | **0.69 (0.57-0.83)** | **0.80 (0.66-0.97)** |  |
| CVD |  |  |  |  | 0.764 |
| Yes | ref | 0.83 (0.66-1.04) | **0.67 (0.52-0.85)** | 0.88 (0.69-1.12) |  |
| No | ref | 0.81 (0.65-1.01) | **0.69 (0.55-0.86)** | **0.75 (0.60-0.93)** |  |
| Cancer |  |  |  |  | 0.856 |
| Yes | ref | 0.77 (0.56-1.05) | 0.76 (0.53-1.10) | **0.71 (0.51-0.98)** |  |
| No | ref | 0.85 (0.71-1.02) | **0.69 (0.57-0.83)** | 0.83 (0.69-1.00) |  |
| CKD stage |  |  |  |  | 0.415 |
| 1 | ref | 0.81 (0.46-1.42) | **0.50 (0.27-0.94)** | 0.65 (0.34-1.25) |  |
| 2 | ref | 1.01 (0.71-1.42) | **0.69 (0.48-0.98)** | 0.78 (0.54-1.12) |  |
| 3 | ref | **0.78 (0.64-0.96)** | **0.65 (0.53-0.80)** | **0.80 (0.65-0.98)** |  |
| 4 | ref | 0.94 (0.52-1.67) | 1.00 (0.56-1.82) | 1.12 (0.57-2.19) |  |
| ACR |  |  |  |  | **<0.001** |
| ≦30mg/g | ref | 0.81 (0.63-1.03) | **0.75 (0.58-0.98)** | 0.91 (0.71-1.17) |  |
| ﹥30mg/g | ref | 0.88 (0.71-1.08) | **0.64 (0.52-0.79)** | **0.76 (0.61-0.94)** |  |
| Hemoglobin |  |  |  |  |  |
| Male: ﹤13.0 g/dL | ref | 0.62 (0.38-1.02) | 0.71 (0.44-1.17) | 0.77 (0.47-1.26) | 0.806 |
| Male: ≧13.0 g/dL | ref | 1.00 (0.78-1.28) | **0.76 (0.59-0.99)** | 0.93 (0.71-1.21) |  |
| Female: ﹤12.0 g/dL | ref | 1.21 (0.64-2.29) | 0.62 (0.30-1.30) | 1.50 (0.77-2.93) | **0.015** |
| Female: ≧12.0 g/dL | ref | 0.79 (0.62-1.02) | **0.67 (0.51-0.86)** | **0.76 (0.60-0.98)** |  |
| Serum phosphorus |  |  |  |  | 0.552 |
| ≦3.05 mg/dL | ref | 1.00 (0.60-1.65) | **0.52 (0.28-0.96)** | 1.04 (0.61-1.76) |  |
| 3.06-4.45 mg/dL | ref | **0.78 (0.65-0.93)** | **0.65 (0.55-0.79)** | **0.77 (0.64-0.92)** |  |
| ﹥4.46 mg/dL | ref | 1.08 (0.61-1.92) | 1.12 (0.65-1.92) | 1.37 (0.77-2.44) |  |

Adjusted covariates: age, sex, family income-poverty ratio level, race, education level, marital status, alcohol consumption, smoking, and leisure-time physical activity, log-transformed total energy intake, HEI-2015, baseline eGFR, log-transformed urinary ACR, body mass index, total-to-HDL cholesterol ratio, serum phosphorus, hemoglobin, hypertension, and diabetes, and history of cardiovascular disease and cancer. The variable used for stratification was not included in the given model. Interaction was tested using a continuous total carotene intake term and the exposure of interest, using a Wald test for dichotomous variables.

*Q* quintile, BMI body mass index, CVD cardiovascular disease, CKD chronic kidney disease, ACR albumin-Cr ratio.


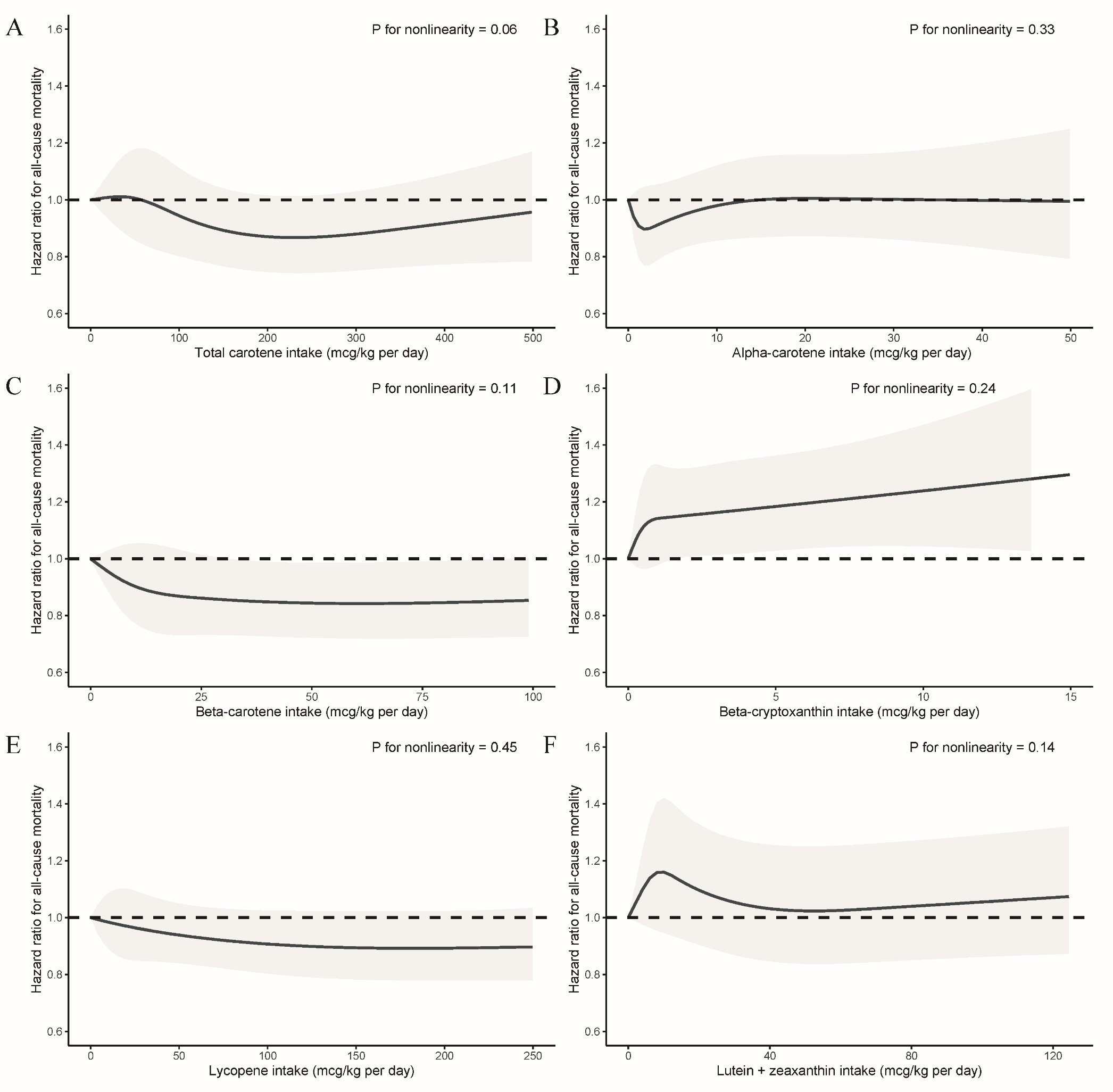


**Supplementary Figure 1** Dose-response analysis of the associations of daily intakes of carotene with mortality. The x-axes show the daily intakes of carotene (mcg/kg/d), and the y-axes show the hazard ratios for mortality risk. The solid curves show hazard ratios compared with the reference levels from restricted cubic spline functions, and the grey zones show 95% confidence intervals of hazard ratios. Restricted cubic spline functions with knots located at the median of each group (i.e., 16.84, 54.65, 113.86 and 297.83 mcg/kg/d for total carotene intake) were applied. Adjusted covariates: age, sex, family income-poverty ratio level, race, education level, marital status, alcohol consumption, smoking, and leisure-time physical activity, log-transformed total energy intake, HEI-2015, baseline eGFR, log-transformed urinary ACR, body mass index, total-to-HDL cholesterol ratio, serum phosphorus, hemoglobin, hypertension, and diabetes, and history of cardiovascular disease and cancer. HDL high density lipoprotein, HEI-2015 healthy eating index score-2015, eGFR estimated glomerular filtration rate, ACR albumin-Cr ratio.


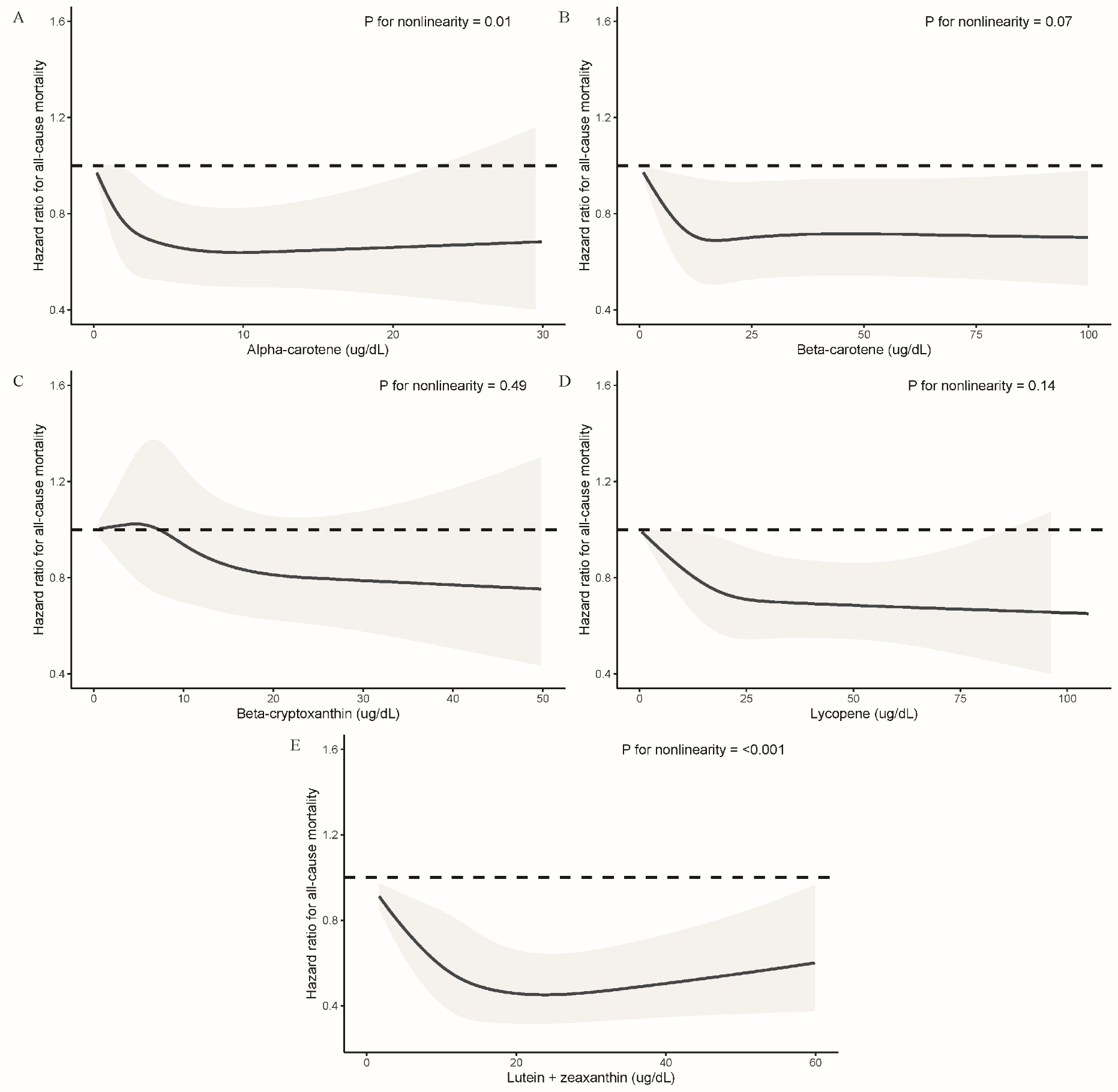


**Supplementary Figure 2** Dose-response analysis of the associations of serum carotenoids with mortality. The x-axes show the serum carotenoids (ug/dL), and the y-axes show the hazard ratios for mortality risk. The solid curves show hazard ratios compared with the reference levels from restricted cubic spline functions, and the grey zones show 95% confidence intervals of hazard ratios. Restricted cubic spline functions with knots located at the median of each group were applied. Adjusted covariates: age, sex, family income-poverty ratio level, race, education level, marital status, alcohol consumption, smoking, and leisure-time physical activity, log-transformed total energy intake, HEI-2015, baseline eGFR, log-transformed urinary ACR, body mass index, total-to-HDL cholesterol ratio, serum phosphorus, hemoglobin, hypertension, and diabetes, and history of cardiovascular disease and cancer. HDL high density lipoprotein, HEI-2015 healthy eating index score-2015, eGFR estimated glomerular filtration rate, ACR albumin-Cr ratio.
